# New Glycosylated Polyene Macrolides: Refining the Ore from Genome Mining

**DOI:** 10.3390/antibiotics11030334

**Published:** 2022-03-03

**Authors:** Patrick Caffrey, Mark Hogan, Yuhao Song

**Affiliations:** School of Biomolecular and Biomedical Science, University College Dublin, D04 V1W8 Dublin, Ireland; mark.hogan@ucdconnect.ie (M.H.); yuhao.song@ucdconnect.ie (Y.S.)

**Keywords:** glycosylated polyene macrolide, antifungal antibiotics, biosynthetic gene clusters, genome mining

## Abstract

Glycosylated polyene macrolides include effective antifungal agents, such as pimaricin, nystatin, candicidin, and amphotericin B. For the treatment of systemic mycoses, amphotericin B has been described as a gold-standard antibiotic because of its potent activity against a broad spectrum of fungal pathogens, which do not readily become resistant. However, amphotericin B has severe toxic side effects, and the development of safer alternatives remains an important objective. One approach towards obtaining such compounds is to discover new related natural products. Advances in next-generation sequencing have delivered a wealth of microbial genome sequences containing polyene biosynthetic gene clusters. These typically encode a modular polyketide synthase that catalyzes the assembly of the aglycone core, a cytochrome P450 that oxidizes a methyl branch to a carboxyl group, and additional enzymes for synthesis and attachment of a single mycosamine sugar residue. In some cases, further P450s catalyze epoxide formation or hydroxylation within the macrolactone. Bioinformatic analyses have identified over 250 of these clusters. Some are predicted to encode potentially valuable new polyenes that have not been uncovered by traditional screening methods. Recent experimental studies have characterized polyenes with new polyketide backbones, previously unknown late oxygenations, and additional sugar residues that increase water-solubility and reduce hemolytic activity. Here we review these studies and assess how this new knowledge can help to prioritize silent polyene clusters for further investigation. This approach should improve the chances of discovering better antifungal antibiotics.

## 1. Introduction

**Figure 1:**
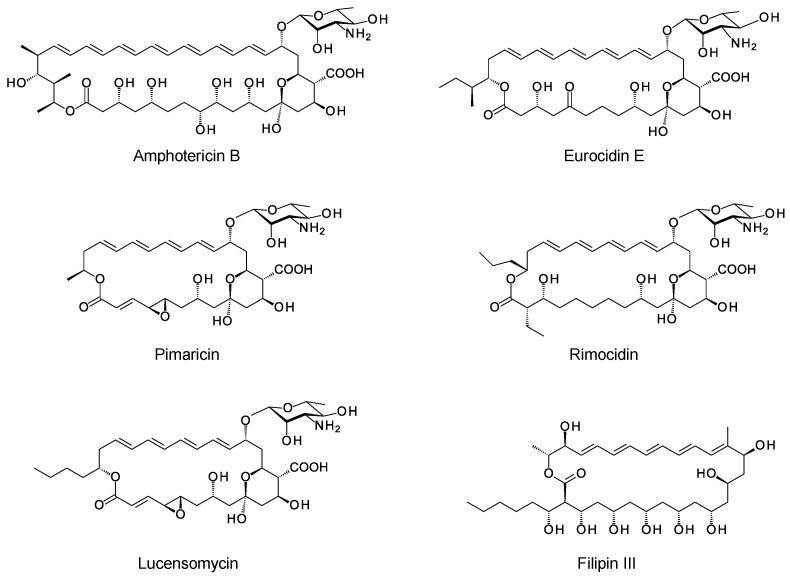
Structures of polyene macrolides.

Polyene macrolides are antifungal agents that are synthesized by actinomycetes and other bacteria [1]. These compounds consist of macrolactone rings containing between three and eight conjugated double bonds (Figure 1). Most are glycosylated with a single aminodeoxysugar and specifically bind ergosterol in fungal cell membranes. Some non-glycosylated pentaenes, such as filipin, interact with cholesterol, are less selective, and disrupt eukaryotic membranes in general. Hundreds of glycosylated polyene macrolides (GPMs) have been identified and chemically characterized, at least partially [2]. A few are used as antifungal antibiotics. One of these, amphotericin B, is the gold standard for treatment of systemic mycoses [3]. It is also used to treat diseases caused by *Leishmania* parasites, which have ergosterol and closely related sterols in their membranes [4].

Polyenes have potent antifungal activity, because binding of ergosterol adversely affects membrane permeability and fluidity, as well as other functions, such as exocytosis and endocytosis, trafficking of nutrient transporter proteins between intracellular and cytoplasmic membranes, and septation at hyphal tips [5]. Amphotericin B resistance has been slow to develop in fungal pathogens even though the drug has been in clinical use for over 60 years [6]. *Leishmania* parasites are less strictly dependent on ergosterol and can resist amphotericin B by synthesizing alternative sterols that do not bind polyenes. In parasites, this resistance mechanism is not associated with a loss of fitness or virulence [7,8,9].

Despite its status as one of the leading antifungal antibiotics, amphotericin B has serious negative aspects. It is not water-soluble, must be given intravenously, and has severe side effects [3]. Liposomal amphotericin B moderates toxicity problems and is a significant improvement over earlier formulations [10]. Amphotericin B analogues with reduced toxicity have been made by chemical modification and by genetic engineering of producer microorganisms [11]. While none of these compounds has been developed into a drug, there is a substantial knowledge of structure–function relationships in glycosylated polyenes and clear evidence that superior analogues exist in the chemical space [12]. One approach towards discovering these compounds is to identify a larger range of producer microorganisms by mining genomes for biosynthetic genes for new polyenes.

Advances in technology are giving access to increasing numbers of actinomycete genome sequences, with a typical genome containing about twenty silent biosynthetic gene clusters (BGCs) for natural products [13]. Analysis of this information is greatly assisted by tools such as antiSMASH and Prism4 [14,15,16], which give automated annotation of BGCs and, in some cases, prediction of the structures of the compounds they encode. The MiBiG database catalogues complete BGCs and correlates sequence data with any experimental characterization that has been reported in the literature [17]. These resources are making an invaluable contribution to discovery of new bioactive compounds. However, accessing the product of a silent BGC is not straightforward. Strategies to overcome the challenges involved have been reviewed by the Muller group [18]. Testing of different growth media may reveal conditions in which the cryptic compound is synthesized. Activation of a cluster requires growth on various media and in different conditions. Genetic manipulation may be required to inactivate repressors, overproduce transcriptional activators, or delete competing clusters [18,19]. Purification, chemical characterization, and assessment of biological activities also require investment of time, effort, and resources. Genome mining is valuable because it rapidly eliminates previously known compounds and helps to prioritize organisms capable of synthesizing the most promising new structures.

In 2018, Liang and co-workers analyzed all publicly available BGCs for glycosylated polyene macrolides. They analyzed evolutionary relationships between PKS and late genes from over 182 different clusters [20]. More recently, Guo and co-workers increased the number of curated polyene BGCs to 252 (104 complete and 148 incomplete clusters) [21].

Here we review the latest experimental studies on glycosylated polyenes, discuss how this new knowledge can be applied to prioritize cryptic BGCs for further investigation, and consider the prospects for obtaining improved antifungal antibiotics.

## 2. Characteristic Features of Polyene BGCs

The macrolactone rings of GPMs are synthesized by modular polyketide synthases. There is now a detailed understanding of how these enzymatic assembly lines select starter and extender units, determine alcohol and methyl stereochemistry, and impose double-bond geometry [22]. Within the multienzyme polypeptides, acyltransferase (AT), ketoreductase (KR), and enoylreductase (ER) domains contain conserved motifs that correlate with the stereochemical outcome of each reaction catalyzed. In some cases, structural studies have given insights into the functions of the key amino acids in the reaction mechanism. Bioinformatic analysis now allows accurate prediction of polyketide stereostructures from amino acid sequences of PKS enzymes [22,23,24,25,26,27]. These prediction methods are a useful aid to chemical methods for determination of polyketide structures in general. Stereostructures predicted from silent polyene PKSs maintain the stereochemical homology found in experimentally characterized GPMs (Figure 1) [11,28].

**Figure 2:**
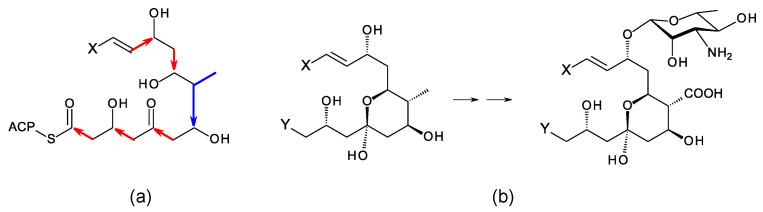
Conserved hemiketal structure that forms in polyene macrolides. (**a**) A hexamodular polyketide synthase protein synthesizes this region of the polyketide chain from malonyl-derived acetate (red arrows) and methylmalonyl-derived propionate (blue arrow) building blocks. (**b**) Exocyclic carboxyl group formation and glycosylation with mycosamine occur in this region. X represents the primer end of the polyketide chain, and Y represents the carboxy-terminal end.

A typical polyene PKS has a series of modules for synthesis of a polyene unit containing at least four conjugated double bonds [29]. This is followed by a hexamodular PKS protein capable of synthesizing a region that forms a hemiketal, includes a methyl branch that is oxidized to a carboxyl group, and a hydroxyl group that is glycosylated with mycosamine (Figure 2) [29]. In the amphotericin PKS, this hexamodular protein is AmphI. The cluster contains genes for a cytochrome P450 (AmphN) that oxidizes the methyl branch, GDP-α-d-mycosamine synthase (AmphDII), and a d-mycosamine-specific glycosyltransferase (AmphDI). A GDP-α-d-mannose 4,6-dehydratase (AmphDIII) functions at an early stage in mycosamine biosynthesis [30]. Homologues of these genes are typical of polyene BGCs. BlastP searches with AmphI, AmphN, and AmphDI can reveal new clusters [20,21]. Additional P450s may modify the polyol chain further [31,32]. These modifications occur at different positions in different polyenes, so it is not possible to predict the precise functions of homologues of these P450s in silent clusters.

There are a few examples of polyenes that lack exocyclic carboxyl groups or are glycosylated with sugars other than d-mycosamine. These are mentioned later in the text. d-mycosamine is not unique to GPMs but also appears in vancoresmycin, a non-polyene macrolide that disrupts membranes of Gram-positive bacteria [33,34]. The vancoresmycin BGC also contains homologues of AmphDIII, AmphDII, and AmphDI.

## 3. Updates on Aromatic Heptaene Biosynthesis

**Figure 3:**
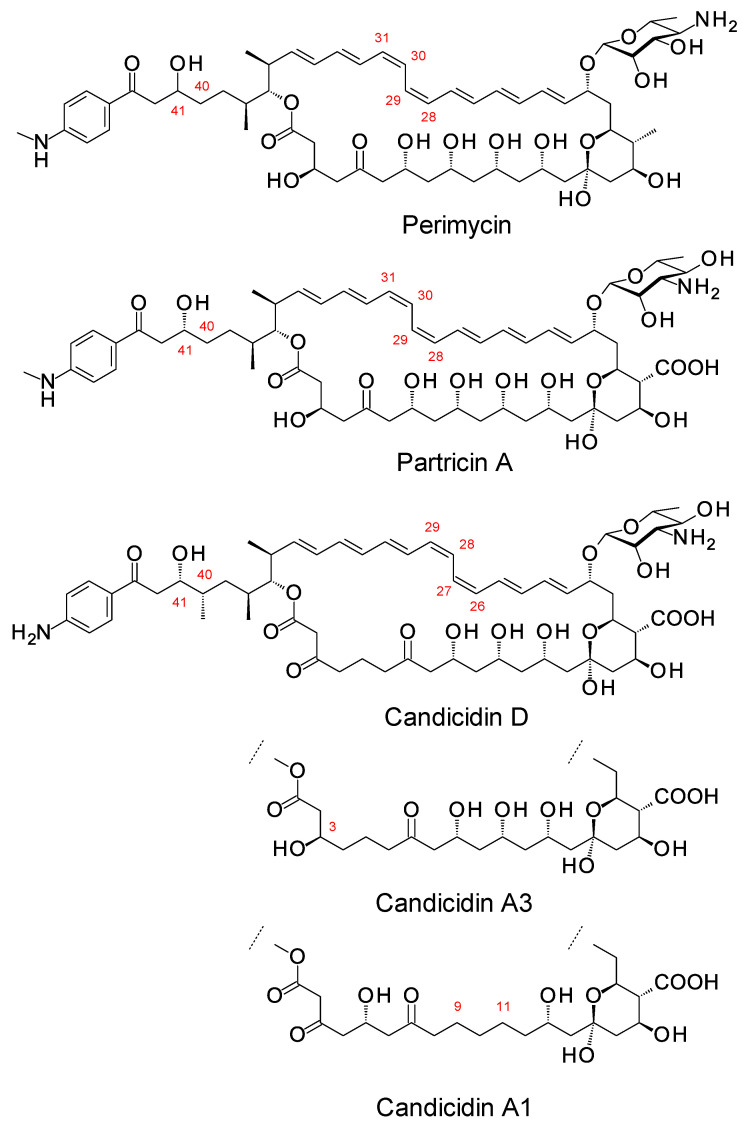
Structures of aromatic heptaenes. Candicidin D = ascocin A2 = levorin A2; candidicin A1 = ascosin A1 = levorin A1; candicidin A3 = ascocin A3 = levorin A3. Partricin B (not shown) is the same as partricin A, except that it lacks an N-methyl group on the *p*-aminobenzoyl moiety. Partricin A and gedamycin are identical, and partricin B and vacidin are identical. Candicidins are synthesized by *Streptomyces albidoflavus* (formerly *Streptomyces griseus*); ascocins are preoduced by *Streptomyces canescus*; levorins are made by *Actinomyces levoris*. Partricins, gedamycin, and vacidin are synthesized by different isolates of *Streptomyces aureofaciens*. Some carbon atoms are numbered to highlight structural differences between the polyenes.

Aromatic heptaenes fall into two subgroups exemplified by candicidins and partricins. These mainly differ in the locations of two consecutive *cis* double bonds in the heptaene region and in the presence or absence of a methyl branch at C40 in the side-chain (Figure 3). Candicidins are produced as a complex of three major components, namely A1, D, and A3. The stereostructures of all three have been determined by NMR spectroscopy [35,36,37]. Stereostructures have also been determined for partricins A and B and perimycin [38,39]. Candicidins have been independently isolated from different microorganisms and given various names [36]. The nomenclature is summarized in the legend in Figure 3. The partricin group consists of polyenes with very similar macrolactone core structures, but some members have unusual late modifications. For example, perimycin has no exocyclic carboxyl group, and the sugar is d-perosamine, not d-mycosamine.

BGCs for aromatic heptaenes occur frequently in actinomycete genomes [40]. Bioinformatic analysis of the PKS sequence can identify the subgroup of the product. In extension module 2, the AT domain is methylmalonate-specific in the candicidin PKS and malonate-specific in the partricin group PKSs. In modules 8 and 9 of the candicidin PKS, DH domains are paired with A-type KR domains. This leads to formation of *cis* double bonds (C26–C27 and C28–C29) [41,42]. In partricin group PKSs, modules 7 and 8 contain DH-KR_A_ pairings. This accounts for the different locations of the two *cis* double bonds in the polyene unit (C28–C29 and C30–C31). Most DH domains are paired with B-type KRs and give *trans* alkenes, which occur more frequently in polyketide natural products.

In candicidin polyketide biosynthesis, the last five cycles are catalyzed by a tetramodular PKS protein containing modules 17 to 20 and a single module protein containing module 21 and the chain-terminating thioesterase (TE). Variable β-ketone processing by modules 17, 18, and 21 results in the formation of candicidins D, A1, and A3. In biosynthesis of partricin group polyketides, a bimodular protein catalyzes cycles 17 and 18 and a trimodular protein with a terminal TE domain catalyzes cycles 19, 20, and 21, and final macrolactone formation [43]. This PKS group appears to synthesize a single type of polyketide chain.

**Figure 4:**
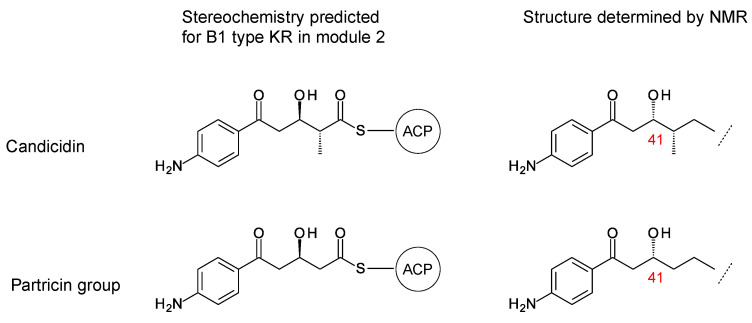
Conflict between structures determined by NMR spectroscopy and by bioinformatic predictions from aromatic heptaene PKS KR2 sequence motifs.

The stereostructures deduced from aromatic heptaene PKSs agree with those determined by NMR, except for the C41 hydroxyl group. Both candicidin and partricin PKSs contain B1-type KR domains in module 2, with clear LDD motifs. The candicidin PKS module 2 is predicted to synthesize a (2*R*,3*R*)-2-methyl-3-hydroxyacyl-ACP2 intermediate, whereas the partricin module 2 should synthesize a (3*R*)-3-hydroxyacyl-ACP2 intermediate. The final stereochemistry predicted for C41 differs from that deduced by interpretation of the NMR data (Figure 4). To our knowledge, this is a unique example of a stark conflict between structures obtained by prediction and NMR spectroscopy. Otherwise, there is agreement between predicted and experimentally determined chirality and double-bond geometry.

Controlled UV irradiation gives specific and irreversible conversion of aromatic heptaenes to all *trans* forms [44,45]. This reduces hemolytic activity and improves selective toxicity [46].

Some members of the partricin group are synthesized as complexes in which a proportion of the total polyene is N-methylated on the *p*-aminobenzoyl moiety. This methylation increases antifungal activity [47]. Sheehan et al. overproduced the AceS N-methylase from *Couchioplanes caeruleus*, producer of the 67-121 complex of aromatic heptaenes [43]. Methylase activity was tested in vitro against surrogate substrates. The recombinant protein catalyzed SAM-dependent methylation of 4-aminobenzoyl-butyl ester but did not act on *p*-aminobenzoic acid. This suggests that the methylase acts at some point after the *p*-aminobenzoate starter has been incorporated into a polyketide chain. Within producer cells, this strict substrate specificity ensures that the methylase does not antagonise folate biosynthesis, an indispensable primary metabolic pathway that requires *p*-aminobenzoic acid and is a major target for antibacterial and antiparasitic drugs [48]. Heterologous expression of AceS N-methylase in *Streptomyces albidoflavus* did not result in the formation of methylated candicidins in vivo [43].

The aromatic heptaenes are of interest because of their high antifungal activity. Derivatives of partricins A and B make up mepartricin, the active pharmaceutical ingredient of the drug Ipertrofan, which is used as a treatment for benign prostatic hyperplasia [38]. A disaccharide-modified aromatic heptaene 67-121C is of interest because it has increased water-solubility (see below).

## 4. Silent Polyene PKSs Predicted to Use a Wider Range of Starter and Extender Units

For the well-studied polyene antibiotics, polyketide biosynthesis is primed with a limited range of starter units. Acetyl primers are used in the biosynthesis of amphotericin, nystatin, and pimaricin; a propionyl primer is used for selvamicin and lucensomycin; a butyryl primer is used for rimocidin; and *p*-aminobenzoate is used for aromatic heptaenes [11,41,49]. An analysis of the recently sequenced *Streptomyces eurocidicus* genome indicates that the eurocidin PKS uses (2*S*)-2-methylbutyrate as a primer [21]. An analysis of BGCs in genome sequences indicates that primers such as guanidinobutyrate, 3-amino-5-hydroxy-benzoic acid, and 3-hydroxybenzoic acid may be used by some polyene PKSs. Some of these PKSs are predicted to use ethylmalonyl and methoxymalonyl CoA extender units, which diversify macrolactone structures further. This section describes some specific examples. Genome accession numbers and isolation sources of microorganisms discussed are given in Supplementary Materials Table S1. The supplementary figures contain more detail on how polyene structures were predicted. The Supplemantary Excel Files S1 and S2 contain tables of genes from the various clusters and PKS motifs used for stereostructure prediction. 

### 4.1. Guanidinobutyrate Primers

**Figure 5:**
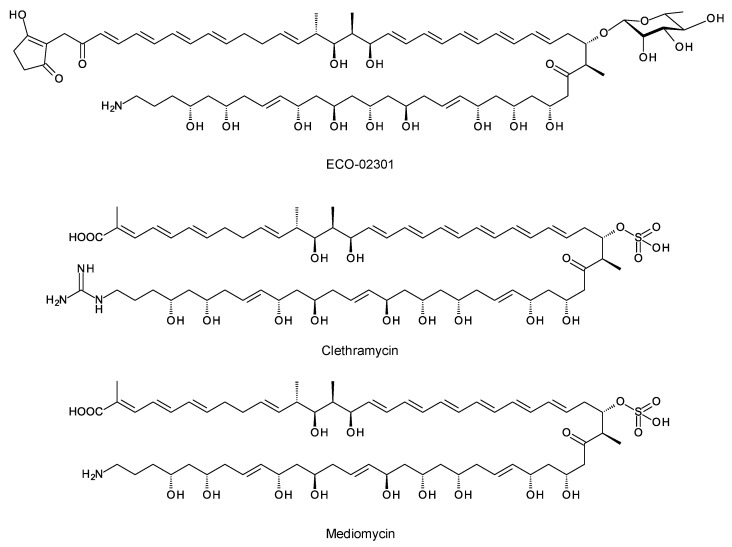
Structures of ECO-02031, clethramycin, and mediomycin.

**Figure 6:**
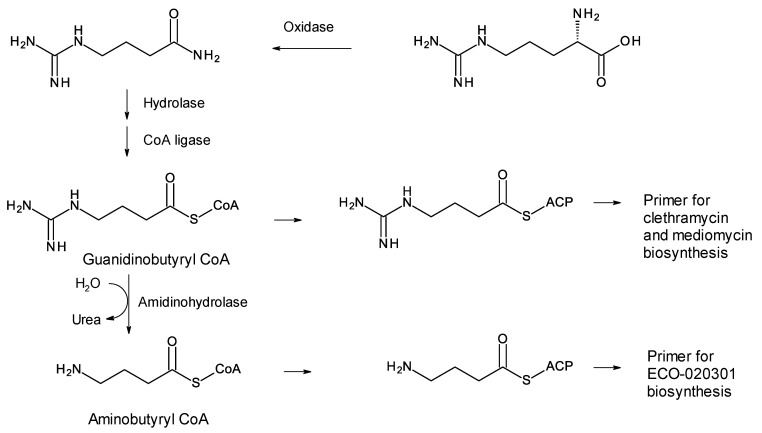
Formation of primers for biosynthesis of linear polyene polyols ECO-020301, clethramycin, and mediomycin.

A few polyketides are known for which biosynthesis of the polyketide chain begins with arginine-derived guanidinobutyryl or 4-aminobutyryl primers (Figure 5). These include antifungal linear polyene polyols ECO-02301 from *Streptomyces aizunensis* NRRL B-11277 [50], clethramycin from *Streptomyces malaysiensis* DSM4137, and mediomycin from *Streptomyces mediocidicus* [51,52,53] (Figure 5). Three enzymes convert l-arginine to guanidinobutyryl CoA: a decarboxylating arginine mono-oxygenase, an amide hydrolase, and a CoA ligase [51] (Figure 6). The ECO-020301 cluster also contains a gene for an amidinohydrolase that cleaves the guanidinobutyryl CoA so that a 4-aminobutyryl chain migrates onto the loading ACP to initiate polyketide biosynthesis (Figure 5 and Figure 6). An intact guanidinobutyryl unit acts as a primer for biosynthesis of clethramycin and mediomycin (Figure 6). In *S. malaysiensis*, the guanidinobutyryl primer remains in the final polyene, clethramycin. *S. mediocidicus* initially synthesizes the same polyene, but the guanidino group is hydrolyzed as a late modification, releasing urea and leaving a 4-aminobutyryl moiety as the remainder from the starter unit. The final product is mediomycin A1. The amidinohydrolase involved (Medi4948) is encoded by a gene located 670 kb distant from the mediomycin BGC in the *S. mediocidicus* chromosome [52].

**Figure 7:**
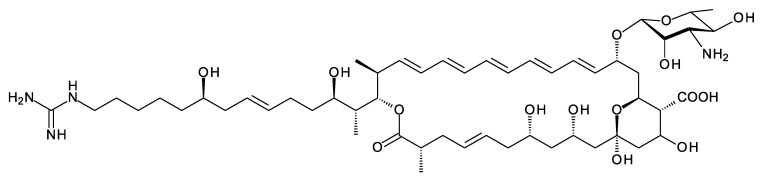
Predicted partial structure of the *A. saalfeldensis* polyene. The structure-prediction method used is given in the 2017 paper by Sheehan et al. [43].

*Amycolatopsis saalfeldensis* has a polyene BGC that includes homologues (57% identical and 71% similar) of the amine oxidase and acyl CoA synthetase that generate guanidinobutyryl CoA as a primer. The PKS assembly line starts with a loading ACP homologous to that for azalomycin PKS, which also uses a guanidinobutyryl primer [53] (Supplementary Materials Figures S1–S3). The predicted polyene structure is shown in Figure 7. It is not possible to predict whether the guanidinobutyryl starter is cleaved after incorporation. The purification and characterization of these polyenes would be necessary to determine the structures correctly. Related BGCs are present in the genomes of *Amycolatopsis jejuensis* and *Amycolatopsis benzoatilytica*, but these are incompletely sequenced.

So far, it has not been possible to detect the production of these polyenes in cultures of *Amyc. saalfeldensis* and *Amyc. benzoatilytica* [54]. These pentaenes are of interest because the presence of an amino or guanidino group may increase antifungal activity. In general, a net-positive charge increases the antimicrobial activity of a biocide that acts on membranes [55]. This may result from initial electrostatic interactions between the negatively charged microbial cell surfaces and positively charged surfactants.

### 4.2. Primer: 3-Amino-5-hydroxybenzoate

AHBA, 3-Amino-5-hydroxybenzoic acid, is used in the biosynthesis of several natural products. It serves as a primer for biosynthesis of ansamycin polyketides such as rifamycin and chaxamycin [56,57,58]. In the producers of these polyketides, AHBA is synthesized from kanosamine 6-phosphate in a complex pathway that requires seven enzymes [56].

**Figure 8:**
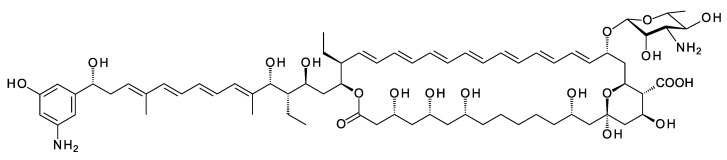
Predicted partial structure of *Amyc. albispora* heptaene (see also Supplementary Materials Figure S4). The BGC contains a gene for an AmphL cytochrome P450 homologue that may hydroxylate the polyol chain between C2 and C14. No prediction is made for this modification.

*Amycolatopsis albispora* was found in a deep-sea sediment sample taken from the Indian Ocean [59]. The genome has a polyene BGC that includes genes for biosynthesis of AHBA from kanosamine [56], and the PKS loading module starts with a CoA ligase domain that is predicted to activate AHBA (Supplementary Materials Figures S4 and S5). AntiSMASH analysis predicts that extension modules 6 and 8 use ethylmalonyl CoA as extender unit but genes for biosynthesis of this extender are absent from the cluster. Our structure prediction for the *Amyc. albispora* heptaene is shown in Figure 8 (see also Supplementary Materials Figures S4 and S5). The extra amino group might increase activity. *Amycolatopsis* YIM10 has a similar cluster that is incompletely sequenced [60].

### 4.3. Starter: 3-Hydroxybenzoate

**Figure 9:**
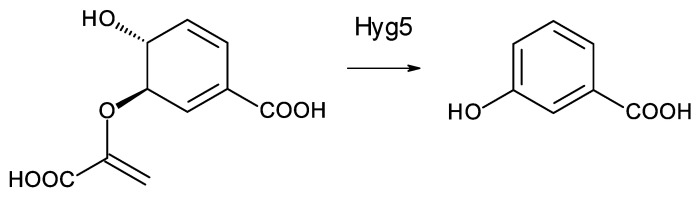
Conversion of chorismate to 3-hydroxybenzoate catalyzed by Hyg5. Homologues of Hyg5 occur in the polyene BGCs of *Sacc. flava* (WP_093416095.1), *Sacc. dendrathemae* (WP_145741796.1), *Lentz. Waywayandensis* (WP_093588251), and *Lentz. Xingjiangensis* (WP_089951167.1).

Two *Saccharopolyspora* species and two *Lentzea* species are predicted to synthesize similar heptaenes that are primed with 3-hydroxybenzoyl units. The *Saccharopolyspora dendrathemae* genome has a complete BGC for an all *trans* heptaene (Supplementary Materials Figures S6 and S7). A similar BGC in the genome of *Saccharopolyspora flava* has been described by Usachova [61]. The sequence of this cluster has not been disclosed but an independently determined incomplete version of the genome sequence for *Sacc. flava* DSM 44771 is publicly available (accession number = NZ_FOZX01000003). The PKSs start with CoA ligase domains that are predicted to activate a substituted benzoic acid as primer for polyketide chain initiation. Similar incomplete polyene PKS assembly lines occur in *Lentzea xinjiangensis* and *Lentzea waywayandensis* DSM44232. All four microorganisms lack genes for AHBA biosynthesis but have a gene for Hyg5 chorismatase that converts 9horismite to 3-hydroxybenzoate (Figure 9) [61,62,63].

**Figure 10:**
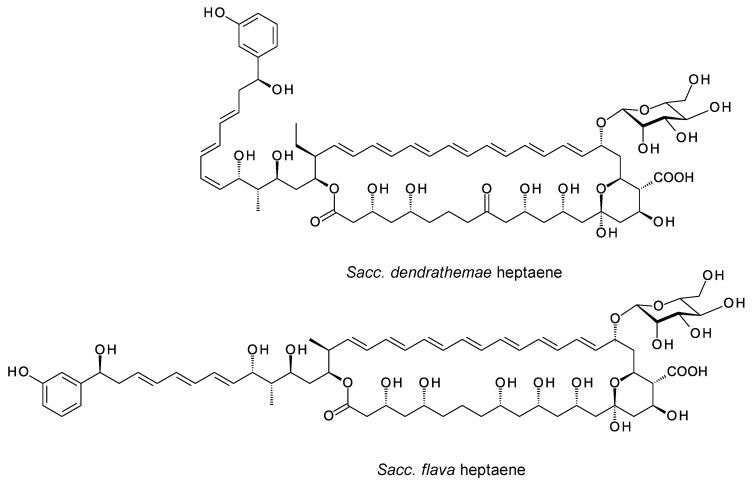
Predicted partial structures of heptaenes made by *Sacc. dendrathemae* and *Sacc. flava*. The two clusters each contain genes for AmphN homologues that form the exocyclic carboxyl group and two further cytochrome P450s for which functions cannot be predicted. The *Sacc. dendrathemae* polyene structure was predicted as detailed in Supplementary Materials Figures S6 and S7. The *Sacc. flava* structure was predicted by Usachova [61].

The Leadlay group overproduced the *Sacc. flava* chorismatase and reconstituted 3-hydroxybenzoate formation in vitro [61]. The *Sacc. dendrathemae* and *Sacc. flava* clusters both encode glycosyltransferases (GTs) homologous to AmphDI (62% identity), but there are no GDP-mannose DH or mycosamine synthase genes in the genomes of either microorganism. AmphDI is capable of using GDP-α-d-mannose as an alternative activated sugar donor [64], so these polyenes are possibly glycosylated with d-mannose. Alternatively, they may be synthesized as aglycones. Predicted structures of the two heptaenes from *Sacc. dendrathemae* and *Sacc. flava* are shown in Figure 10.

Usachova demonstrated production of the *Sacc. flava* heptaene, named flavatericin, but the partially purified material was not amenable to analysis by mass spectrometry. This heptaene had no detectable antifungal activity, as is consistent with the absence of an aminodeoxyhexose sugar. The *Sacc. dendrathemae* and *Sacc. flava* heptaenes may have other biological functions, such as signaling molecules that enable intercellular communication in mixed microbial communities [65].

The PKS genes are not completely assembled for *Lentz. Waywayandensis* and *Lentz. Xingjiangensis*. Manual curation of the fragments suggests that both clusters could encode 24-module PKSs capable of synthesizing all-trans heptaenes similar to flavatericin. The two *Lentzea* BGCs have AmphDIII and AmphDII homologues, indicating that the macrolactones are glycosylated with mycosamine or perosamine.

**Figure 11:**
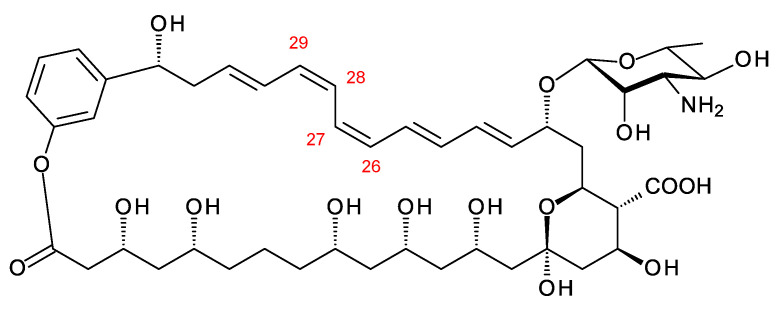
Ps2 phylotype polyene. This structure is the same as that predicted by Holmes and co-workers [46], except for the geometry of the C26–C27 and C28–C29 double bonds. In the PKS extension modules 3 and 4, DH domains are paired with A-type KR domains, indicating that *cis* alkenes are formed. The genome used for this structure prediction has the accession number MCIQ00000000.

Moreover, 3-Hydroxybenzoate is predicted to serve as a primer for another class of glycosylated polyene. Holmes and co-workers identified Ps1 and Ps2 phylotypes of *Pseudonocardia* symbionts of ants [66]. The Ps1 phylotype strains synthesize disaccharide-modified nystatins (see below). The genomes of five Ps2 phylotype strains contain a BGC for a pentaene polyketide that is primed with a substituted benzoic acid residue. The cluster contains a Hyg5 family chorismatase (protein accession OLM23048.1) that is predicted to synthesize 3-hydroxybenzoate. The PKS starts with a CoA ligase that is predicted to activate a substituted benzoic acid. The structure predicted for the product of this BGC is shown in Figure 11. So far, it has not been possible to detect production of this pentaene [66].

## 5. Meijiemycin and Related Linear Polyene Polyols

**Figure 12:**
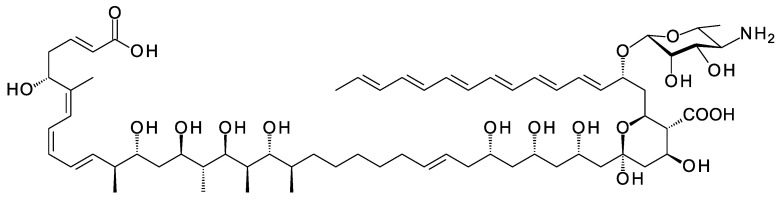
Structure of meijiemycin.

A new class of glycosylated polyene is synthesized by *Streptomyces* SD50, an organism found in a marine sediment sample taken from a mangrove swamp in Singapore [67]. Meijiemycin is a linear polyene polyol (Figure 12) that is glycosylated with d-perosamine (4-amino-4,6-dideoxy-d-mannose). The biosynthetic genes are weakly expressed in wild-type *Streptomyces* SD50. Low and co-workers [67] increased production by inactivating two competing clusters and by growing the double mutant in the presence of mannitol, thought to boost formation of GDP-α-d-mannose, a perosamine precursor. The mode of action of meijiemycin was investigated by treating *Candida albicans* cells with an Alexafluor 488-labeled derivative. Examination by fluorescence microscopy revealed that the polyene localized in ergosterol-rich membrane microdomains and impaired formation of hyphal filaments from yeast cells [67]. Meijiemycin has a minimum inhibitory concentration (MIC) of 12 ± 4 μg/mL, a lower antifungal activity than cyclized polyene macrolides [67]. However, preventing the yeast to hyphal switch may abolish virulence without killing the fungal cell [68].

**Figure 13:**
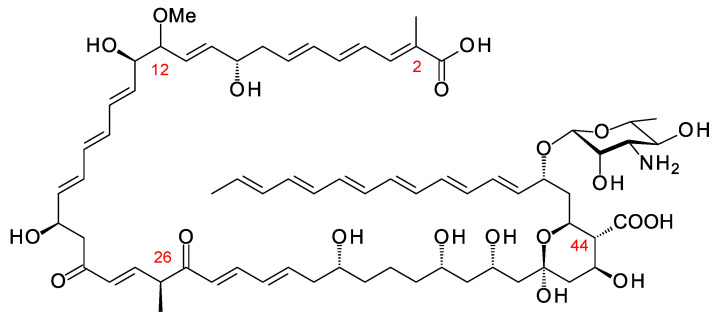
Predicted partial structure of *Amyc. lexingtonensis* polyene. Methyl branches are shown at C2 and C44 as predicted by antiSMASH, although the relevant domains AT29 and AT8 have FAAH motifs, such as the methoxymalonate-specific AT24, not YASH motifs, such as methylmalonate-specific ATs. AT17 has YASH, and so module 17 is predicted to introduce the methyl branch at C26.

*Amycolatopsis lexingtonensis* has a BGC predicted to specify a hexaene polyol related to meijiemycin (Figure 13; Supplementary Materials Figures S8 and S9). AntiSMASH predicts that the polyketide chain contains methyl branches at C2, C26, and C44, and a methoxy branch at C12. AT24 introduces the C12 methoxy branch and has FAAH in place of the YASH or HAFH motifs characteristic of methylmalonate and malonate-specific AT domains. AT8 and AT29 also have FAAH motifs, raising the possibility that one or both of these domains use methoxymalonyl extenders to form methoxy branches at C44 and C2. The cluster does encode an AmphN homologue (64% identity), which would oxidize a methyl branch at C44. The genome of *Amycolatopsis eburnea* contains an incompletely sequenced cluster that is closely related to this hexaene-polyol BGC from *Amyc. lexingtonensis*.

*Amyc. lexingtonensis* has a second set of AmphDIII-DI-DII homologues associated with a BGC for a non-polyene polyketide related to vancosresmycin, which is also glycosylated with d-mycosamine [33].

*Streptomyces milbemycinicus*, *Streptomyces bingchengensis*, and *Streptomyces* 14.10 all have incomplete BGCs closely related to that for meijiemycin, as does a metagenomic DNA sequence from *Streptomyces* isolate Bin7.12.2. All of these clusters include genes for proteins that are >96% identical to the MjmSII GDP-perosamine synthase, indicating that the polyenes are glycosylated with d-perosamine.

Incomplete BGCs for meijiemycin-related polyene polyols are also present in genomes of other actinomycetes, namely *Streptosporangium album* DSM 43023, *Acrocarpospora macrocephala*, and *Acrocarpospora pleiomorpha*. Manual curation of the fragmented sequences indicates that these clusters specify structures that are slightly different from meijiemycin. The clusters from the two *Acrocarpospora* species have genes for dTDP-glucose 4,6-DH and an additional GT that is not homologous to any GT of known function.

## 6. Kineosporicin/Actinospene

**Figure 14:**
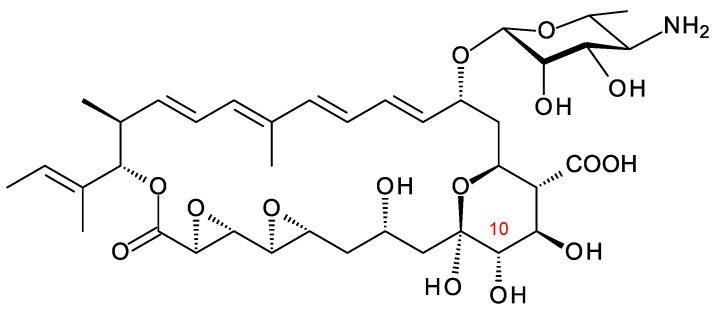
Kineosporicin/actinospene from *A. spheciospongiae*.

*Actinokineospora spheciospongiae* DSM45935 synthesizes a methyltetraene that is glycosylated with d-perosamine, contains two epoxide groups, and is also unusual in that it is hydroxylated at C10 (Figure 14). This tetraene has been characterized independently by two groups and named kineosporicin [21] and actinospene [69]. The *Actino. spheciospongiae* BGC is closely related to a complete cluster in *Actinokineospora mzabensis*. *Actino.* *spheciospongiae* was isolated from a sponge in the Red Sea, Egypt [70], whereas *Actino.* *mzabensis* was obtained from a sample of Saharan soil taken from South Algeria [71].

The *Actino. spheciospongiae* cluster contains genes for four cytochrome P450 enzymes. One is an AmphN homologue that forms the exocyclic carboxyl group. The other three are likely to form the two epoxides and hydroxylate C10. It is not yet known which P450 catalyzes each of these reactions. Actinospene was active against yeasts and filamentous fungi and had MICs comparable to those of pimaricin (2 to 10 μg/mL). The diepoxide and the extra C10 hydroxylation apparently do not increase antifungal activity but could have other positive effects on other pharmacological properties [21,69].

## 7. Enzymes Involved in Synthesis and Attachment of Mycosamine or Perosamine

**Figure 15:**
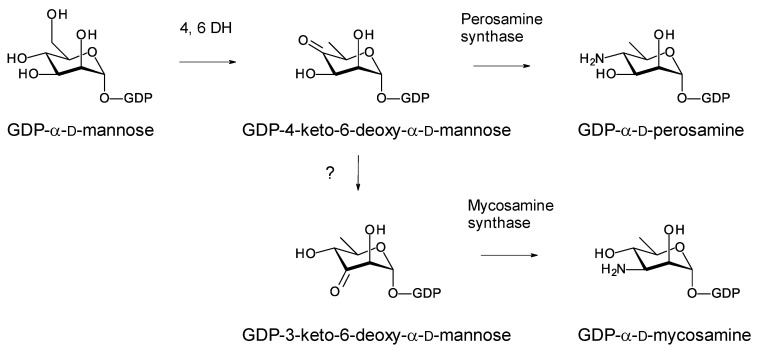
Biosynthetic pathways for GDP-α-d-perosamine and GDP-α-d-mycosamine.

Most glycosylated polyene macrolides are modified with a d-mycosamine sugar. A few are modified with d-perosamine. Perosamine also occurs in the lipopolysaccharides of some Gram-negative pathogens. The biosynthesis of GDP-α-d-perosamine from GDP-α-d-mannose has been reconstituted in vitro and occurs in two steps: GDP-α-d-mannose 4,6-dehydratase catalyzes the formation of GDP-4-keto-6-deoxy-α-d-mannose, and perosamine synthase catalyzes transamination [72] (Figure 15). It has been proposed that GDP-α-d-mycosamine formation involves isomerization of GDP-4-keto-6-deoxy-α-d-mannose to GDP-3-keto-6-deoxy-α-d-mannose, followed by a transamination catalyzed by mycosamine synthase (Figure 15). There are several 3,4-ketoisomerases that act on dTDP-4-keto-6-deoxy-α-d-glucose, but no counterpart has been identified that acts on GDP-4-keto-6-deoxy-α-d-mannose. Spontaneous 3,4-ketoisomerization of GDP-4-keto-6-deoxy-α-d-mannose occurs slowly in vitro [73], suggesting that this step in mycosamine biosynthesis proceeds in the absence of an enzyme in vivo. GDP-α-d-mycosamine is a precursor for biosynthesis of vancoresmycin, a non-polyene macrolide. The vancosresmycin biosynthetic gene cluster contains AmphDIII and AmphDII homologues but also lacks a candidate 3,4-ketoisomerase gene [33]. Fluviricin macrolactam antibiotics contain l-mycosamine, the mirror image stereoisomer of d-mycosamine [74]. Biosynthesis of dTDP-α-l-mycosamine from dTDP-4-keto-6-deoxy-α-d-glucose also requires a 3,4-ketoisomerization step for which no gene or enzyme has been identified. These observations amount to circumstantial evidence that the 3,4-ketoisomerization is not enzyme-catalyzed. However, so far, it has not been possible to synthesize GDP-α-d-mycosamine from GDP-α-d-mannose in vitro, using GDP-α-d-mannose 4,6-dehydratase and GDP-α-d-mycosamine synthase enzymes [75].

Four classes of polyene macrolides are now known that are glycosylated with perosamine. These are perimycin, meijiemycin, kineosporicin/actinospene, and a pimaricin analogue named JB1R-13 [76]. The sequence of the BGC for JB1R-13 is not available. The BGCs for perimycin, meijiemycin, and kineosporicin provide three classes of verified GDP-α-d-perosamine synthases that function in GPM biosynthesis: (1) PerDII (Accession ADD39188.1), (2) MjmSII (Accession AYW76484.1), and (3) *Actinokineospora spheciospongiae* perosamine synthase (Accession WP_035287523.1). Databases contain two transaminases that are >99.72% identical to PerDII and four that are >96% identical to MjmSII.

At the primary-sequence level, these three classes of polyene GDP-α-d-perosamine synthases show 69 to 71% sequence identity to each other and are 69 to 73% identical to AmphDII, a GDP-α-d-mycosamine synthase. The alignment of these three polyene GDP-perosamine synthases with known GDP-mycosamine synthases revealed no obvious motifs or conserved residues specific for either group (unpublished results). The polyene ketosugar aminotransferases show only low homology (36 to 40% sequence identity) to perosamine synthases that function in lipopolysaccharide biosynthesis in Gram-negative bacteria [72].

Perosamine- and mycosamine-specific glycosyltransferase sequences show 57 to 62% identity, with no characteristic differences in the C-terminal GDP-sugar binding domain [77]. Primary sequence homology is not useful for bioinformatic prediction of whether a BGC specifies a polyene glycosylated with mycosamine or perosamine. The Mitchell group has devised a pHMM model for predicting whether a polyene aminosugar is perosamine or mycosamine [21]. In the case of amphotericin B, replacing the mycosamine with perosamine made little difference to antifungal activity in vitro [77].

## 8. Disaccharide-Modified Polyenes

Medicinal chemistry has shown that the addition of a second sugar to the mycosamine residue contributes to improvements in the pharmacological properties of amphotericin B and other polyene macrolides [78]. In dimethylformamide, the aldehyde group of glucose efficiently condenses with the amino group of mycosamine to form an imine that rearranges to an N-fructosyl analogue. A few naturally occurring polyenes have a second sugar residue linked to C4′ of mycosamine [41].

### 8.1. A Disaccharide-Modified Aromatic Heptaene, 67-121C

**Figure 16:**
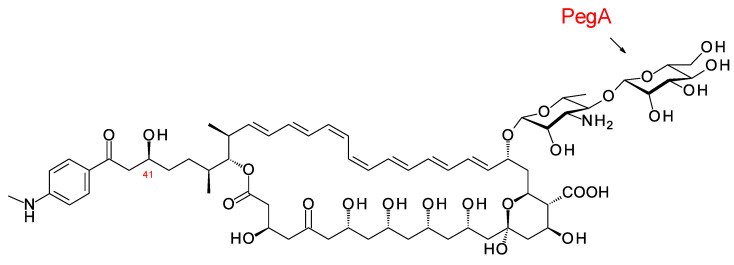
Structure of 67-121C.

The disaccharide-modified aromatic heptaene 67-121C (Figure 16) is produced by *Couchioplanes caeruleus* DSM43634, formerly known as *Actinoplanes caeruleus*. A predicted stereostructure for the 67-121 polyketide [43] showed agreement with the experimentally determined stereostructures for members of the partricin group, except for the C41 hydroxy group (see above).

The polyene extending glycosyltransferase PegA adds the second mannosyl sugar to the mycosamine residue of the aromatic heptaene 67-121A to give the disaccharide-modified form, 67-121C [79] (Figure 16). The function of the enzyme was confirmed by heterologous expression of the *pegA* gene in the candicidin producer *Streptomyces albidoflavus*, which gave low but detectable yields of mannosyl-candicidins [43,80]. The *pegA* gene is not present in the 67-121 BGC but is located at a distance of 23,437 bp along the chromosome within a transposable element. The genome of *Actinoplanes digitatis* has a cluster identical to the 67-121 BGC but lacks an extending glycosyltransferase (EGT) gene.

### 8.2. Disaccharide-Modified Nystatins

**Figure 17:**
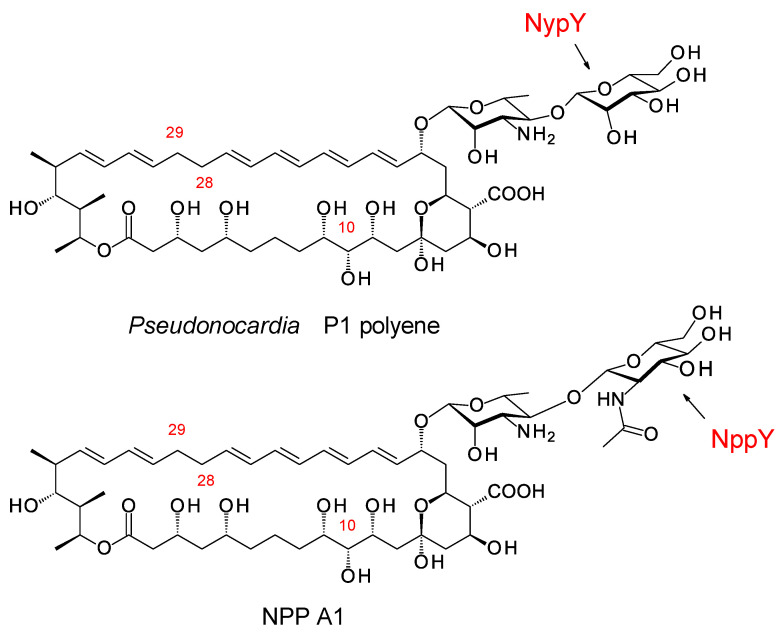
Disaccharide-modified *Pseudonocardia* polyenes.

Groups in the UK and Korea discovered *Pseudonocardia* species that synthesize two different disaccharide-modified analogues of nystatin (Figure 17) [81,82]. In both analogues, the second sugar is attached to C4′ of mycosamine. *Pseudonocardia* P1 is an ant symbiont that synthesizes a nystatin in which the second sugar is mannose, added by the NypY extending GT [81]. *Pseudonocardia autotrophica* KCTC9441 synthesizes NPP A1 (nystatin-like *Pseudonocardia* polyene), in which the second sugar is N-acetylglucosamine (GlcNAc). Compared to nystatin A1, NPP A1 has a 2-fold decrease in antifungal activity but is 10 times less aemolytic and 300 times more water-soluble [82]. The extending GT is NppY. During NPP A1 biosynthesis, the addition of the second sugar precedes the final late modification, hydroxylation of the macrolactone at C10 [83]. The NppL P450 catalyzes this reaction with the disaccharide-modified 10-deoxynystatin as substrate. In *Streptomyces noursei*, the original nystatin A1 producer, the corresponding NysL P450 acts on a monosaccharide-modified 10-deoxytetraene substrate [84].

The NppL and NysL P450s have been expressed in Δ*nppL* and Δ*nppL*-*nppY* mutants of *Ps. autotrophica* [85]. The NppL C10 hydroxylase was apparently specific for disaccharide-modified 10-deoxy macrolactones, whereas *S. noursei* NysL hydroxylated both monosaccharide- and disaccharide-modified 10-deoxy substrates. The in vivo complementation approach was used to assess a series of NppL–NysL hybrid enzymes. This work showed that the C-terminal 50 amino acid residue region of NppL functions in rejection of monosaccharide-modified 10-deoxy substrates [85].

*Ps. autotrophica* KCTC9441 has been genetically engineered extensively. This work has delivered four disaccharide-containing analogues of NPP A1: (1) a 10-deoxy tetraene, NPP A2 [86]; (2) a heptaene with a double bond between C28 and C29, NPP B1 [87]; (3) a 10-deoxy heptaene, NPP B2 [88]; and (4) NPP A3, a tetraene in which mannose replaces the GlcNAc sugar [89]. The 10-deoxy tetraene and the heptaene NPP B1 had increased antifungal activity. The 10-deoxyheptaene NPP B2 had good activity and significantly reduced hemolytic activity. Replacing the GlcNAc of NPP A1 with mannose was detrimental, as the NPP A3 analogue was inferior in terms of antifungal activity and hepatotoxicity [89]. This indicates that the structure of the second sugar is important.

Yields of the potentially improved NPP B1 and NPP B2 analogues were increased by strain improvement of the producer organisms. This was achieved by nitrosoguanidine mutagenesis and screening for increased production, and by eliminating a 128 kb plasmid carrying a BGC for a competing polyketide. In addition, an integrating plasmid was used to introduce an extra copy of the 32 kb region containing positive regulatory genes. These measures increased the yields to over 31 mg per liter of production culture for NPP B1 [90] and 7 mg per liter for NPP B2 [88].

### 8.3. Identification of New Polyene Extending GTs by Genome Mining

Homologues of NypY and NppY occur in genomes of several *Pseudonocardia* species. A protein identical to NppY is encoded within the genome of *Ps. autotrophica* DSM43083, which was sequenced because of its lignolytic activity [91]. NppY is also encoded within the genome of *Pseudonocardia* SID8383, which was sequenced as part of a project aimed at characterizing antibiotic-producing symbionts of ants [92]. Two slightly different proteins that are both 99% identical to NppY are encoded within the genomes of an endophyte *Pseudonocardia alni* [93] and an Antarctic isolate *Pseudonocardia antarctica* [94]. Another ant symbiont, *Ps* AL041005-10, has an enzyme that is 89% identical to NppY [95].

Proteins that are >99% identical to the mannose-specific NypY occur in the genomes of several ant symbionts. An identical protein is encoded within *Ps.* Ae707Ps1 [66]. Genes for a protein with 99% sequence identity occur within the genomes of *Pseudonocardia* strains ECO80610-09 and ECO80619-01 [96]. A slightly different protein, also 99% identical to NypY, is encoded within the genomes of four other strains, namely Ae150Aps1, Ae168Ps1, Ae263Ps1, and Ae356Ps1 [66].

In summary, six *Pseudonocardia* genomes have a gene for a protein that is at least 89% identical to NppY, and eight genomes have a gene for a protein that is at least 99% identical to NypY (Supplemantary Materials Table S2). All of these GTs are encoded by genes in nystatin BGCs. Alignment of NppY with NypY reveals 83% sequence identity. Alignment of the variants of these enzymes reveal few differences between *N*-acetylglucosamine-specific and mannose-specific enzymes (Supplementary Materials Figure S10). Extending GTs share about 50% sequence identity with their corresponding mycosaminyltransferases, but sequence alignments reveal clear sequence differences between the two groups (Supplementary Materials Figure S10). Some amino acid residues and motifs characteristic of NppY and NypY are also conserved in PegA. During manual curation of a polyene BGC, it is possible to distinguish between an enzyme that mycosaminylates a polyene aglycone and an extending GT that adds a second sugar to the mycosamine. We have found two new GT sequences that align with PegA (Supplementary Materials Figure S11). These are encoded within polyene BGCs in *Cryptosporangium arvum* DSM 44712 and *Amycolatopsis suaedae*.

**Figure 18:**
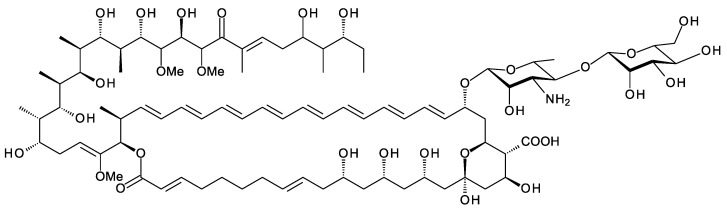
Predicted structure for octaene from *Cryptosporangium arvum*.

*Crypto. arvum* DSM 44712 is a Japanese soil isolate [97] containing a BGC for an octaene (Figure 18; Supplementary Materials Figures S12 and S13). Two of the PKS genes have frameshifts that may be sequencing errors or real mutations. The cluster includes a gene for a putative extending GT (EXG82143.1) that is homologous (49% identical and 64% similar) to PegA (WP_071803650.1). This polyene is of interest because the Zotchev group found that a minor octaene analogue of nystatin had high antifungal activity [98].

**Figure 19:**
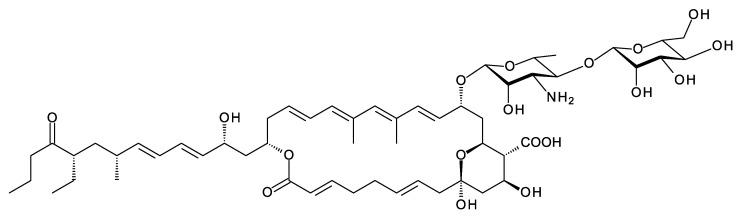
Partial structure predicted for *Amyc. suaedae* tetraene. The BGC contains genes for a AmphN P450 homologue (63% identity) that forms the exocyclic carboxyl group and genes for two further cytochrome P450 enzymes (WP_130475496.1 and WP_130475500.1) homologous to SelL (47% identity) and Lcm10 (47% identity). The modifications catalyzed by these enzymes cannot be predicted. Lcm10 catalyzes epoxide formation in lucensomycin. There are also genes for biosynthesis of additional deoxyhexoses and two further GT genes (see Section 10).

*Amyc. suaedae* is an endophyte obtained from a *Suaeda maritima* salt marsh plant collected in Thailand [99]. The *Amyc. suaedae* genome has a BGC for a methyl tetraene with a long side-chain (Figure 19; Supplemenatry Materials Figures S14 and S15). An AmphDI homologue (WP_1304475493.1, 55% identity) is likely to add the mycosamine residue. There is a gene for an extending glycosyltransferase (WP_130478880.1) that is 59% identical to PegA. This is predicted to mannosylate the mycosamine at C4′. The cluster contains genes for biosynthesis and attachment of additional deoxysugars (see below), suggesting that the real polyene may be more extensively glycosylated than the hypothetical core structure in Figure 19.

### 8.4. Extending GTs in Synthetic Biology

Early studies have investigated the potential of extending glycosyltransferases (EGTs) as tools in synthetic biology. NypY has been expressed in the amphotericin producer, *Streptomyces nodosus*, and in engineered strains that synthesize various analogues [80]. NypY gave modest mannosylation of amphotericins A and B and 7-ketoamphotericins A and B, but not the less toxic analogues in which a methyl group replaces the exocyclic carboxyl group. Expression in *S. nodosus* Δ*amphL* gave mannosyl-8-deoxyamphotericins A and B [100]. The yields of these analogues were higher than for C8-hydroxylated disaccharide-modified forms. This suggests that NypY is specific for 10-deoxynystatins and 8-deoxyamphotericins but does not efficiently glycosylate forms that are hydroxylated at C10 or C-8. Expression of NypY in *S. noursei* did not give disaccharide-modified NPP A1 [101], possibly because the native pathway rapidly C-10 hydroxylates and exports the 10-deoxynystatin A1 substrate before NypY has time to act.

Mannosyl-8-deoxyamphotericin B has been purified and characterized by NMR spectroscopy. The second sugar gave no improvement in in vitro antifungal activity compared to 8-deoxyamphotericin B, and a slight reduction in hemolytic activity [100].

NypY shows a greater tolerance towards unnatural acceptor substrates than PegA. NypY acts on candicidins, as well as some amphotericins, whereas PegA acts on candicidins but not amphotericins [80]. These studies indicate that extending GTs have potential for synthesizing disaccharide analogues of amphotericins, candicidins, and possibly other polyenes, such as pimaricin.

## 9. Polyenes Modified with Two Unlinked Monosaccharides

A few naturally occurring polyenes contain d-mycosamine and another monosaccharide that is located at the opposite end of the macrolactone [2]. The second glycosylation does not greatly reduce antifungal activity and increases water-solubility [98]. The effects on other pharmacological properties have not been assessed. This section summarizes what is known about these “two-monosaccharide” polyenes. In the following section, we discuss cryptic BGCs that may encode new polyenes glycosylated with mycosamine and other deoxysugar residues.

### 9.1. Addition of a Second Monosaccharide at the Position Corresponding to C35 of Nystatin

**Figure 20:**
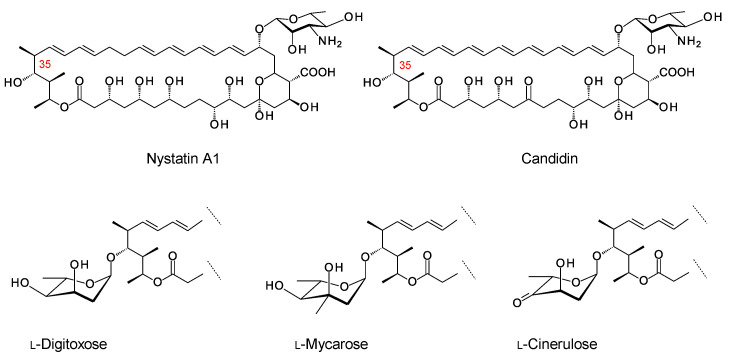
Structures of nystatin A1 and candidin. Modification of nystatin A1 and candidin at C35 with l-digitoxose gives nystatin A3 and candidinin, respectively [76]. Modification of candidin with l-cinerulose gives candidoin. Nystatin analogues modified with l-mycarose have also been identified [98].

The older literature describes analogues of nystatin and candidin that are glycosylated on the C35 hydroxyl group (Figure 20) [102,103,104]. *Streptomyces noursei* synthesizes nystatin A1 as a main product, along with smaller amounts of nystatins A2 and A3. The structure of nystatin A2 has not been determined. Nystatin A3 has an l-digitoxosyl residue attached to the C35 hydroxyl. Polyfungins are made by *Streptomyces noursei* var. *polifungini* ATCC 21581. Polyfungins A1, A2, and A3 appear to be identical to nystatins A1, A2, and A3 [105,106]. The heptaene candidin is produced by *Streptomyces viridiflavus* [78], along with two analogues, candidinin and candidoin, that have l-digitoxose and l-cinerulose, respectively, at C35. In a more recent study, the Zotchev group found that *S. noursei* produces minor nystatin analogues with l-mycarose at C35 [98]. These mycarosylated glyco-analogues were purified from *S. noursei* and from *S. noursei* ERD44, which makes the heptaene analogue of nystatin A1. The mycarose residue caused a slight decrease in antifungal activity for nystatin and its heptaene analogue. However, this extra sugar should increase water-solubility and might have other positive effects on pharmacological properties. The ERD44 mutant also produced octaene analogues of nystatin that seem to result from iterative action of one of the PKS modules that synthesizes the polyene unit. The octaene had a high antifungal activity. A mycarosylated form of the octaene was detected by using LC–MS.

The genome of the candidin producer *Streptomyces viridiflavus* has not been sequenced as yet. The genome sequence of *S. noursei* reveals separate biosynthetic pathways for l-mycarose and l-digitoxose that are encoded within two different BGCs. The l-mycarose biosynthetic genes occur within a cluster for an erythromycin analogue, whereas the l-digitoxose biosynthetic genes occur within a cluster encoding an NRPS for a non-alpha polyamino acid (NAPAA) related to ε-poly-l-lysine. Both clusters contain GT genes, but the identity of the *S. noursei* enzyme that catalyzes transfer of digitoxose or mycarose to the C35 hydroxyl of nystatin is not obvious. The erythromycin cluster in *S. noursei* is active and is the likely source of dTDP-β-l-mycarose [107].

### 9.2. Selvamycin

**Figure 21:**
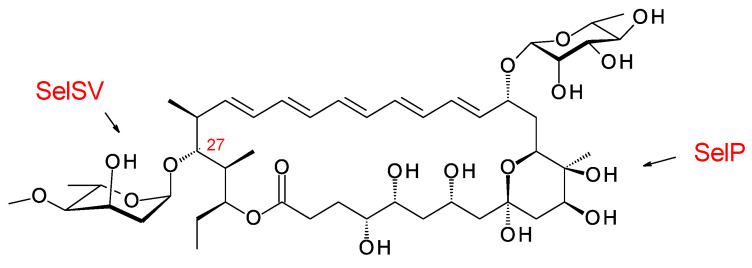
Structure of selvamicin.

Clardy and co-workers isolated the pentaene selvamycin (Figure 21) from *Pseudonocardia* symbionts of ants [108]. Selvamicins are glycosylated with a neutral sugar, d-rhamnose, in place of d-mycosamine. There is no exocyclic carboxyl group, but the carbon atom bearing the relevant methyl branch is hydroxylated by a 2-ketoglutarate-dependent dioxygenase, SelP.

**Figure 22:**
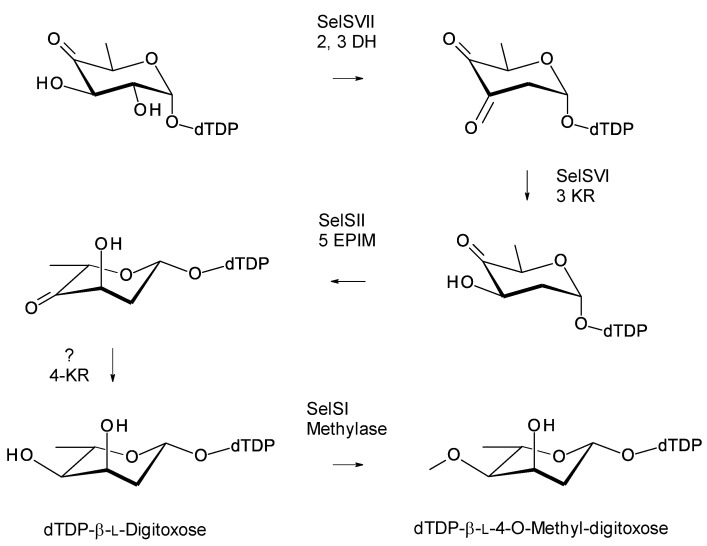
Biosynthesis of dTDP-β-l-4-O-methyl-digitoxose in selvamicin-producing *Pseudonocardia* species. It is possible that methylation occurs after transfer of the l-digitoxosyl residue to the macrolactone. The abbreviations used are as follows: 2,3-DH, dTDP-4-keto-6-deoxyhexose 2,3-dehydratase; 3 KR, dTDP-3,4-diketo-2,6-dideoxyhexose 3-ketoreductase; 5 EPIM, dTDP-4-keto-2,6-dideoxyhexose C5 epimerase; 4KR, dTDP-4-keto-2,6-dideoxyhexose 4 ketoreductase.

The C27 position (corresponding to the C35 OH of nystatin) is glycosylated with 4ʹʹ-O-methyl l-digitoxose. Two *Pseudonocardia* isolates were found to have almost identical selvamicin BGCs: one of these clusters was chromosomal and the other was located in a large plasmid. Both clusters contained all but one of the biosynthetic genes for dTDP-4-O-methyl-l-digitoxose (the 4KR is missing) (Figure 22). The SelSV GT modifies the C27 OH.

Selvamicin has weak ergosterol-binding activity [21] and an MIC of 23 μM, as compared to 1 μM for nystatin A1 [108]. The presence of rhamnose in place of mycosamine is consistent with this reduced activity. However, selvamicin is soluble in water up to 2.3 mM, whereas nystatin A1 is insoluble at concentrations greater than 0.3 mM [108]. The reduced ring size of the pentaene and the extra digitoxosyl sugar may account for the high water-solubility.

The discovery of selvamicin is important because it provided the SelSV protein sequence as a probe for bioinformatic identification of glycosyltransferases that modify the hydroxyl corresponding to C35 of nystatin or other sites within the macrolactone core.

## 10. BGCs for Polyene Macrolides with Additional Glycosylation

This section describes a number of polyene BGCs that include genes for enzymes possibly involved in biosynthesis and attachment of extra deoxysugars and aminodeoxysugars to polyketide macrolactones. We attempt to assign functions to the various proteins based on homology to enzymes of known function. In most cases, the combination of sugar biosynthetic enzymes could give several products. Rather than drawing all of the possible deoxysugar pathways, we make just one “best guess” proposal. The aim is to explain why these clusters are of interest and could be prioritized for further investigation. Each predicted polyene structure should be treated as a hypothesis for testing, not as a serious attempt to determine chemical structure.

### 10.1. Additional Polyene Glycosyl Transferases

Manual curation has revealed eight microorganisms with the potential to synthesize polyenes with sugars additional to the standard mycosamine/perosamine (Table 1). All eight strains have a mycosaminyltransferase and between one and three additional GTs each. Seven of the strains have a SelSV homologue, and three strains have a second AmphDI homologue (51 to 53% sequence identity). Four strains have a homologue (between 47 and 64% identity) of LndGT4, an extending GT that adds the third l-rhodinosyl sugar to the d-olivosyl-d-olivosyl-l-rhodinosyl trisaccharide of the angucycline antibiotic landomycin E, and the third and sixth l-rhodinosyl sugar residues of the d-olivosyl-d-olivosyl-l-rhodinosyl-d-olivosyl-d-olivosyl-l-rhodinosyl hexasaccharide of the related landomycin A [109]. This information is summarized in Table 1. Two of these clusters (*Amycolatopsis antarctica* and *Actinophytocola algeriensis*) contain a gene for a family 39 GT that uses undecaprenol-phosphomannose as sugar donor (not included in Table 1). All eight clusters contain biosynthetic genes for extra NDP-deoxysugars. Some of the sequences are incomplete but the characteristics of a polyene BGC are present. Each case is briefly described below.

### 10.2. Pseudonocardia endophytica

*Pseudonocardia endophytica* is an endophyte of the Chinese medicinal plant *Lobelia clavata* [110]. The *Ps. endophytica* genome has a nystatin BGC that is complete, except for two frameshifts in PKS genes. The cluster includes a gene for a GT with 64% sequence identity to SelSV and biosynthetic genes for dTDP-l-digitoxose. These are similar to those in the selvamicin cluster. These are homologues of SelSIII glucose-1-phosphate thymidylyltransferase (76% identity), SelSVII NDP-4-keto-6-deoxyglucose 2,3-DH (75% identity), Sel VI 3KR (62% identity), and SelSII 5′ epimerase (75% identity). There is no 4KR gene, as is the case with the selvamicin cluster. The dTDP-glucose 4,6-DH gene is also missing from the *P. endophytica* cluster, but not the selvamicin cluster. The *Ps. endophytica* cluster is predicted to encode nystatin A3 (Figure 20), which has an l-digitoxose residue on C35.

### 10.3. Saccharopolyspora gloriosae

**Figure 23:**
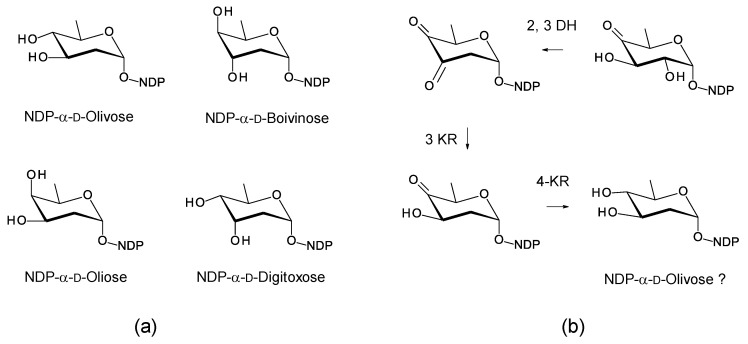
Proposed pathway for biosynthesis of NDP-α-d-2,6-dideoxyhexose in *Sacc. gloriosae*. The combination of genes present could give any one of the four possibilities shown in panel (**a**). NDP-α-d-olivose is predicted in (**b**), because the 2,3-DH (MBB5070950.1) is 67% identical to SelSVII, the 3KR (MBB5070951.1) is 58% identical to SelSVI, and the 4KR (MBB5070952.1) is 47.6 % identical to LanR (AAD13548) [108,112,113].

**Figure 24:**
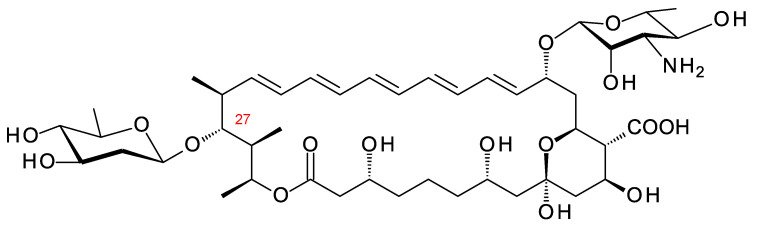
Predicted partial structure for *Sacc. gloriosae* pentaene. The site for glycosylation with d-olivose is predicted to be C27 because the homology between SelSV and MBB5070953.1 is 60% identity. *Sacc. gloriosae* has a SelL homologue (58% identity) that is likely to hydroxylate the polyol chain. This modification is not shown.

*Saccharopolyspora gloriosae* is an endophyte of another Chinese medicinal plant, *Gloriosa superba* [111]. The *Sacc. gloriosae* genome has a PKS that is predicted to synthesize a pentaene similar to the macrolactone of selvamicin (Supplementary Materials Figures S16 and S17). The cluster includes biosynthetic genes for a dTDP-linked neutral 2,6-dideoxy-d-sugar (Figure 23). Four of these dideoxysugars are possible (Figure 23a). The 3KR is 58% identical to SelSVI [108], and the 4KR is 47.6% identical to LanR [112]. These enzymes give the C3′ and C4′ stereochemistry shown in Figure 23b [108,113]; thus, on this basis, dTDP-α-d-olivose is proposed as the product. The *Sacc. gloriosae* cluster includes a gene for a GT (MBB5070953.1) that is 60% identical to SelSV. The predicted partial structure of the pentaene is shown in Figure 24.

### 10.4. Amycolatopsis suaedae

**Figure 25:**
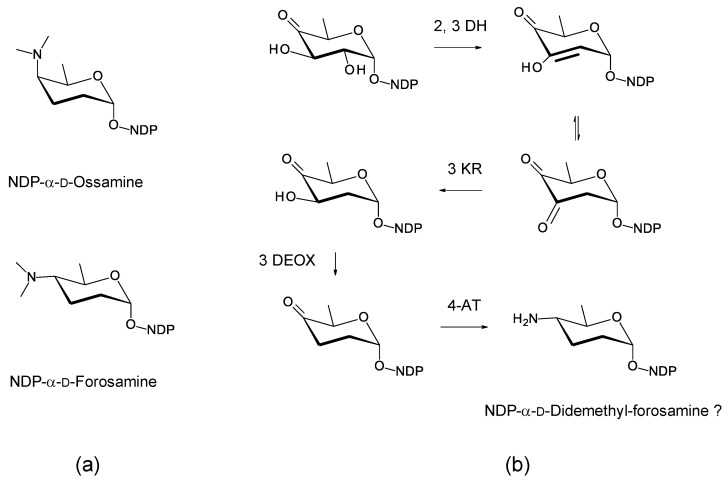
Proposed biosynthesis of dTDP-4-amino-2,3,4,6-tetradeoxy-α-d-glucose in *Amyc. suaedae*. The enzymes encoded could give unmethylated forms of either NDP-α-d-ossamine or NDP-α-d-forosamine (**a**). NDP-α-d-forosamine is predicted because the 4-aminotransferase is 52% identical to VinF, which gives the stereochemistry shown in (**b**). The 2,3-DH (WP_130478887.1) is 62% identical to SelSVII, the 3 KR (WP_165436470.1) is 53% identical to SelSVI, the 3 DEOX (WP_130478889.1) is 70% identical to UrdQ [116], and the 4-AT (WP130479013.1) is 52% identical to VinF [115]. The new abbreviations are as follows: 3 DEOX, NDP-4-keto-2,6-dideoxyhexose 3-deoxygenase; 4-AT, NDP-4-keto-2,3,6-trideoxyhexose 4 aminotransferase.

*Amyc. suaedae* [99] has already been mentioned because it contains a tetraene BGC that includes a homologue of the *pegA* gene for an extending glycosyltransferase (Figure 19). The *Amyc. suaedae* cluster also contains biosynthetic genes for an NDP-2,3,4,6-tetradeoxy-4-amino-d-hexose sugar, NDP-didemethyl-d-ossamine, or NDP-didemethyl-d-forosamine (Figure 25) [113,114]. The latter of these is favored because the 4-aminotransferase is 52% identical to VinF [115], an enzyme of known function. The cluster contains genes for two further GTs, a second AmphDI homologue (WP_130478878.1, 52% identical to AmphDI) and a SelSV homologue (WP_130478888.1, 48% identical to SelSV). One of these GTs might add the extra aminosugar, but the site where another sugar might be attached cannot be predicted. The purification and characterization of this pentaene are required to find out whether extra sugars are present. This example is of interest, because activity might be increased by an additional positively charged sugar and a long side-chain (Figure 19). The cluster also contains a homologue of PegA that may add another hexose to the mycosamine residue.

### 10.5. Amycolatopsis cihanbeyliensis

**Figure 26:**
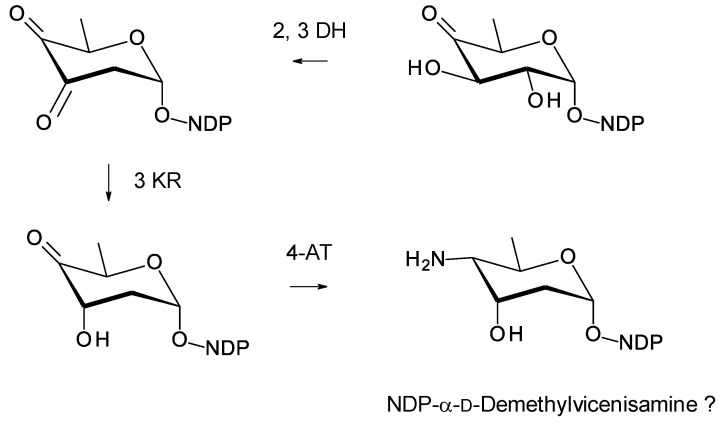
Proposed pathway for biosynthesis of NDP-N-demethyl-α-d-vicenisamine in *Amycolatopsis cihanbeyliensis*. The 2,3-DH (WP_141995336.1) is 59% identical to ScaDH1 [118], the 3KR (WP_141995334.1) is 67% identical to VinE [115] and the 4AT (WP_141995335.1) is 66% identical to VinF [115].

*Amyc. cihanbeyliensis* is a halotolerant actinomycete from the Cihanbeyli salt mine in the central Anatolia region of Turkey [117]. The genome sequence includes a polyene BGC that has one large gap. There is enough information to predict the structure of an advanced intermediate in biosynthesis of the polyene polyketide chain (Supplementary Materials Figures S18 and S19). *Amyc. cihanbeyliensis* contains sugar biosynthetic genes homologous to those for biosynthesis of dTDP-α-d-vicenisamine in *Streptomyces halstedii* [115]. However, there is no N-methylase gene so possibly the sugar is an unmethylated form of d-vicenisamine (Figure 26). Two SelSV homologues are present, WP141995329.1 (51% identical) and WP_141995331.1 (52% identical).

### 10.6. Crossiella cryophila

**Figure 27:**
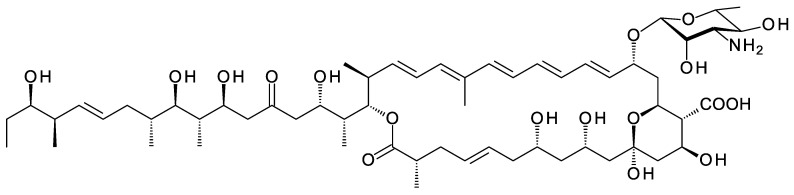
Predicted partial structure for *Crossiella* pentaene.

**Figure 28:**
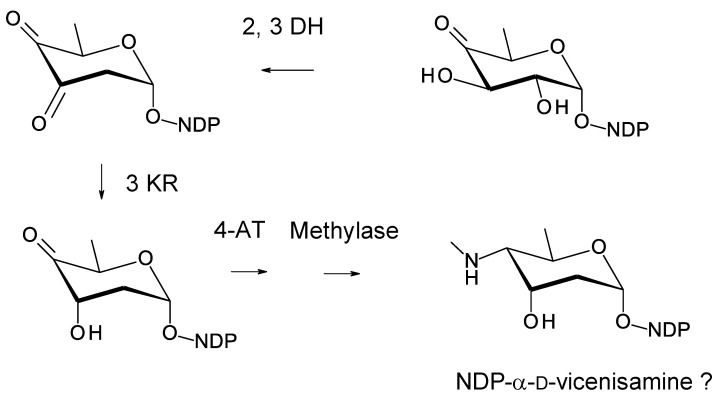
Proposed pathway for biosynthesis of d-vicenisamine in *Crossiella cryophila*. The 2,3-DH (WP_185005856.1) is 62% identical to SelSVII, the 3KR (WP_185005854.1) is 66% identical to VinE, the 4AT (WP_185005851.1) is 62 % identical to SpnR, and the methylase (WP_185005852.1) is 47% identical to SpnS [115,120].

*Crossiella cryophila* DSM44230 was isolated from a Japanese soil sample [119]. The genome has a BGC that specifies a methylpentaene with a side-chain (Figure 27; Supplementary Materials Figures S20 and S21). There are linked genes for mycosamine synthase and mycosaminyltransferase (WP_185005835.1, 56% identical to AmphDI) at one end of the cluster. At the other end, there are genes that could specify d-vicenisamine (2,3-DH; 3KR; 4-aminotransferase, N-methylase) (Figure 28). There is a second AmphDI homologue (WP_185005855.1, 51% identical to AmphDI) and another GT (WP_185005853.1) that is 44% identical to the LndGT4 extending GT. It is possible that this polyene is glycosylated with a d-vicenisamine residue, but the site of attachment cannot be predicted.

### 10.7. Amycolatopsis antarctica

**Figure 29:**
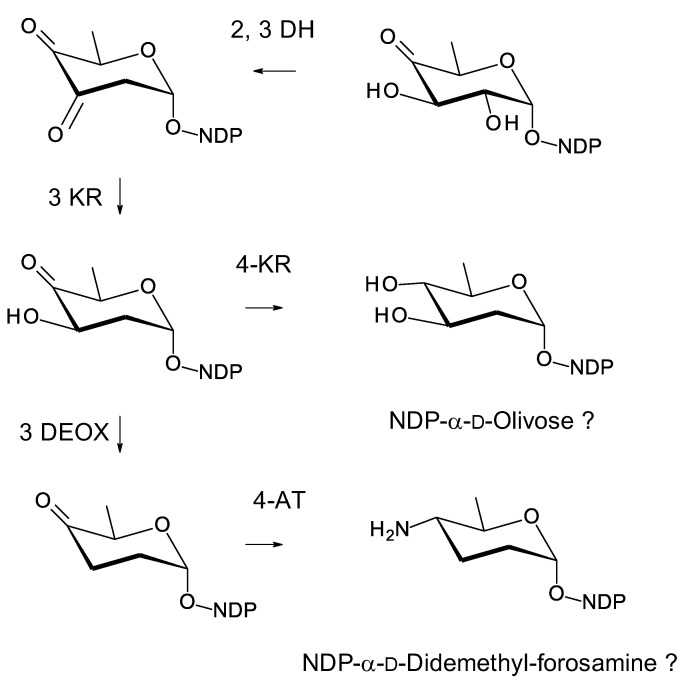
Proposed biosynthesis of NDP-α-d-olivose and NDP-didemethyl-α-d-forosamine in *Amyc. antarctica*. The 2,3-DH (OZM74152.1) is 61% identical to SelSVII, the 3KR (OZM74153.1) is 51% identical to SelVI, the 4KR (OZM74154.1) is 53% identical to Nbc15 [122], the 3DEOX (OZM74156.1) is 74 % identical to IdnS12 [123], and the 4AT (OZM74157.1) is 67% identical to IdnS13 [123]. IdnS12 and IdnS13 function in biosynthesis of dTDP-N-demethylforosamine in *Streptomyces* ML694-90F3, producer of the macrolactam incednine.

*Amyc. antarctica* was isolated from an Antarctic seaweed sample [121]. This genome has an incompletely sequenced polyene BGC. There is one pentamodular polyene PKS protein capable of synthesizing the mycosaminylation site. This is similar to the hexamodular AmphI protein, except that the sixth module is absent (Supplementary Materials Figure S22). There are biosynthetic genes for GDP-α-d-mycosamine and an AmphDI mycosaminyltransferase (OZM74150.1). In addition, there are genes for deoxysugar biosynthetic enzymes that can be fitted to a branched pathway for NDP-linked neutral and aminodeoxysugars, possibly NDP-α-d-olivose and NDP-α-d-didemethylforosamine (Figure 29).

Two genes for extra glycosyltransferases are present: a SelSV homologue (OZM74158.1, 48% identity) and an LndGT4 homologue (OZM74155.1, 45% identity). There is also a family 39 GT (OZM74158.1), which is predicted to use polyprenylphosphate-mannose as activated sugar donor rather than a nucleotide-linked sugar.

Although the sequence is incomplete, the available information indicates that this polyene BGC is worth investigating further.

### 10.8. Actinophytocola algeriensis and Actinophytocola xanthii

**Figure 30:**
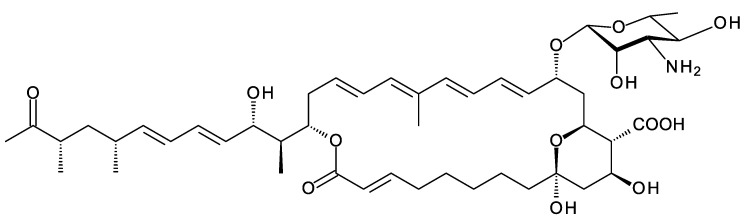
Partial structure predicted for *Actino. algeriensis* tetraene. The cluster includes genes for homologues of AmphL and Lcm10 cytochrome P450 enzymes, the exact functions of which cannot be predicted.

*Actinophytocola algeriensis* was isolated from a Saharan soil sample from Southern Algeria [124]. The genome contains a BGC encoding a complete 17-module PKS for a tetraene with a side-chain (Figure 30; Supplementary Materials Figures S23 and S24). There are “standard late genes”, encoding homologues of AmphDII mycosamine synthase, AmphDI mycosaminyltransferase, AmphN P450, and two other P450s homologous to AmphL C8 hydroxylase and the epoxide-forming Lcm10.

**Figure 31:**
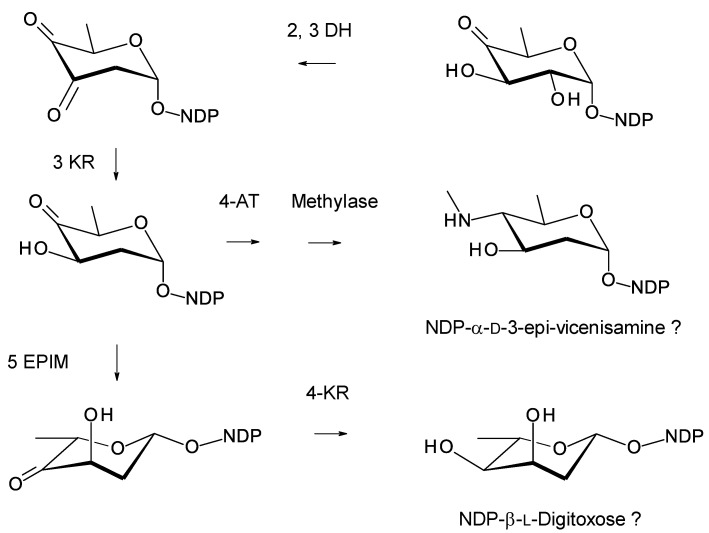
Proposed biosynthesis of d and l sugars in *Actino. algeriensis* and *Actino. xanthii*. In *Actino. algeriensis*, the 2,3-DH (MBB4911558.1) is 61% identical to SelSVII, the 3KR (MBB4911557.1) is 54% identical to SelVI, the 4AT (MBB4911553.1) is 61% identical to SpnR, the methylase (MBB4911552.1) is 50% identical to SpnS, the EPIM (MBB4911555.1) is 57% identical to StaE [126], and the 4KR (MBB4911556.1) is 50 % identical to Nbc15 [122]. In *Actino. xanthii*, the 2,3-DH (WP_075125785.1) is 46% identical to EryBVI, the 3KR (WP_075125784.1) is 59% identical to SelSVI, the 4AT (WP_075125780.1) is 62% identical to SpnR, the methylase (WP_075125779.1) is 52% identical to SpnS, the EPIM (WP_075125782.1) is 54% identical to EryBVII, and the 4KR (WP_075125783.1) is 49% identical to Nbc15 [122].

NDP-deoxysugar biosynthetic genes encode a dTDP-4-keto-6-deoxyglucose 2,3-DH, a 3KR, a 5-epimerase, a 4KR, a 4-aminotransferase, and an N-methylase (Figure 31). This combination of biosynthetic genes could generate at least two deoxysugars. The presence of 4KR and 4-aminotransferase genes suggests that the pathway is branched. Figure 31 makes one proposal based on the sequence homologies between the various enzymes and enzymes of known function.

The same deoxysugar biosynthetic gene subcluster occurs in *Actinophytocola xanthii*, an isolate from rhizosphere soil of the *Xanthium sibiricum* plant from Tangshen, Habei, China [125]. The genome sequence is far from complete (204 contigs), but the deoxysugar cluster is linked to genes characteristic of a polyene BGC.

In *Actino. algeriensis,* there are three additional genes for NDP-deoxysugar-dependent GTs. These are two SelSV homologues, MBB4911547.1 and MBB4911545.1, both 51% identical to SelSV, and one LndGT4 homologue MBB4911554.1 (47% sequence identity). There is also a family 39 GT (MBB4911542.1) that is predicted to use polyprenyl-phosphomannose as activated sugar donor.

The *Actinophytocola xanthii* genome has two AmphDI homologues, OLF06429.1, (59% identity) and WP_075125790.1 (53% identity); a SelSV homologue (WP_O75125789.1, 52% identity); and an LndGT4 homologue (WP_075125781.1, 52% identity).

## 11. Discussion

Advances in next-generation sequencing technology and bioinformatics are delivering an increasing number of BGCs for GPMs. Over 250 have been identified to date. Some of the currently available genome sequences are incomplete or poorly assembled. Manual curation is laborious but can extract valuable information from this low-grade ore. Genome-sequence quality is improving as new technologies are developed. In silico analyses can identify polyene polyketides that differ from the well-characterized tetraenes, pentaenes, heptaenes, degenerate heptaenes, and aromatic heptaenes. “New” structures result from use of a wider range of polyketide starter and extender units, from formation of linear polyene-polyol analogues, and from additional late steps, such as glycosylation and cytochrome P450-catalyzed modifications. NPP A1, *Pseudonocardia* P1 mannosyl-nystatin, selvamicin, meijiemycin, and kineosporicin/actinospene have been purified and characterized relatively recently [21,67,69,81,82,108]. Genome mining indicates that even more new polyenes are encoded by silent clusters. Accessing these compounds requires a considerable investment of time and resources. It is necessary to decide which BGCs should be prioritized for activation and analysis of products. Many of the theoretical polyenes identified by genome mining are predicted to have long side-chains, which may increase antifungal activity. Predictions also suggest that some of these structures are primed with guanidinobutyrate, aminobutyrate, or amino-hydroxybenzoate. These starter units introduce a positively charged groups that can increase the activity of a membrane-active antibiotic [54].

While increased antifungal activity is important, another consideration is reduced toxicity. The presence of an extending glycosyltransferase homologous to NppY, NypY, or PegA suggests that a mycosaminyl residue is glycosylated at C4′ with a hexose sugar. Experimental studies indicate that a disaccharide gives a slight decrease in antifungal activity that is offset by a large increase in water solubility and reduction in hemolytic activity and toxicity. Here we identified two new PegA homologues that are predicted to function in biosynthesis of a disaccharide-modified octaene in *Crypto. arvum* and a methyl pentaene in *Amyc. suaedae*. These clusters are interesting for further experimental characterization.

As well as polyenes modified with mycosaminyl-hexosyl disaccharides, there are polyenes modified with two unlinked monosaccharides, mycosamine and one other deoxysugar that may be l-digitoxose, l-mycarose, or l-cinerulose. With nystatin A3, candidinin, and candidoin, this second sugar is attached to the hydroxyl on C35 of the macrolactone ring. The second monosaccharide causes a slight reduction in antifungal activity in vitro but may improve water-solubility and possibly other pharmacological properties. The discovery of selvamycin has given insights into biosynthesis of these “two-monosaccharide” polyenes. The SelSV is the first identified GT that adds a sugar to a second site within a polyene macrolactone. Genome mining has uncovered nine homologues of SelSV in BGCs for a nystatin and various pentaenes and tetraenes (Table 1). These were accompanied by biosynthetic genes for NDP-deoxysugars and NDP-aminodeoxysugars. Amino deoxysugars related to d-vicenisamine and d-forosamine are predicted for six polyenes. d-olivose is a predicted component of two polyenes, and l-digitoxose is predicted for two others. Predictions are obviously not adequate for determining the structures of deoxysugars, but these observations indicate that some of these clusters should be prioritized and targeted for activation. *Amyc. antarctica* contained a set of genes that could be fitted to a branched pathway generating d-olivose and didemethyl-d-forosamine. *Actinophytocola algeriensis* and *Actinophytocola xanthii* contain the same sub-cluster of deoxysugar biosynthetic genes that can be fitted to a branched pathway leading to l-digitoxose and an epimer of d-vicenisamine. It is possible that these three clusters give polyenes with two extra sugars, as well as mycosamine. *Amyc. suaedae* is also interesting, since it has a polyene cluster containing a gene for an extending GT and genes for biosynthesis and attachment of an aminodeoxysugar, possibly d-forosamine. A second aminosugar on a polyene would be expected to increase both antifungal activity and water-solubility.

Some of these silent BGCs are present in genomes of actinomycetes obtained from soil rather than underexplored environments (Supplementary Materials Table S1). This indicates that new BGCs are cryptic rather than absent, and that terrestrial soil samples have not been exhausted as a source of new antibiotic producers.

In this article, we have attempted to give a perspective on the mass of sequence information currently available for polyene BGCs. While genomes can be rapidly sequenced and automatically annotated, careful analysis and curation of large datasets is essential to identify the strains that are most likely to synthesize new polyene structures. Concentrating attention on potentially rewarding strains should eventually enable access to compounds from which new antifungal antibiotics may be developed.

## Figures and Tables

**Table 1:** Microorganisms containing polyene BGCs with additional polyene glycosyltransferases.

Microorganism	2nd AmphDI Homologue % Identity	SelSV Homologue(s) % Identity	LndGT4 Homologue % Identity
*Ps. endophytica*		WP_165922095.1, 64%	
*Sacc. gloriosa*		MBB5070953.1, 60%	
*Amyc. suaedae*	WP_130478878.1, 52%	WP_130478888.1, 48%	
*Amyc. cihanbeyliensis*		WP_141995329.1, 51%; WP_141995331.1, 52%	
*Crossiella cryophila*	WP_185005855.1, 51%		WP_185005853.1, 47%
*Amyc. antarctica*		OZM74158.1, 52%	OZM74155.1, 64%
*Actino. algeriensis*		MBB4911547.1, 51%; MBB4911545, 51%	MBB4911554.1, 47%
*Actino. xanthii*	WP_075125790.1, 53%	WP_075125789.1, 52%	WP_075125781.1, 52%

## Data Availability

Not applicable.

## Supplementary Materials

The following supporting information can be downloaded at: https://www.mdpi.com/article/10.3390/antibiotics11030334/s1. Figure S1: Domain compositions of *Amyc. saalfeldensis* PKS proteins. Figure S2: Schematic diagram of final polyketide chain made by *Amyc. saalfeldensis* PKS. Figure S3: Structure prediction for macrolactone core of *Amyc. saalfeldensis* polyene. Figure S4: Domain compositions of *Amyc. albispora* PKS proteins. Figure S5: Structure prediction for macrolactone core of *Amyc. albispora* polyene. Figure S6: Domain compositions of *Sacc. dendrathemae* PKS proteins. Figure S7: Structure prediction for macrolactone core of *Sacc. dendrathemae* polyene. Figure S8: Domain compositions of *Amyc. lexingtonensis* PKS proteins. Figure S9: Structure prediction for macrolactone core of *Amyc. lexingtonensis* polyene. Figure S10: Alignment of polyene extending GTs and mycosaminyltransferases. Figure S11: Alignment of PegA and putative extending GTs from *Crypto. arvum* and *Amyc. Suaedae*. Figure S12: Domain compositions of *Crypto. arvum* PKS proteins. Figure S13: Structure prediction for macrolactone core of *Crypto. arvum* polyene. Figure S14: Domain compositions of *Amyc. suaedae* PKS proteins. Figure S15: Structure prediction for macrolactone core of *Amyc. suaedae* polyene. Figure S16: Domain compositions of *Sacc. gloriosa* PKS proteins. Figure S17: Structure prediction for macrolactone core of *Sacc. gloriosa* polyene. Figure S18: Domain compositions of *Amyc. cihanbeyliensis* PKS proteins. Figure S19: Structure prediction for macrolactone core of *Amyc. cihanbeyliensis* polyene. Figure S20: Domain compositions of *Cross. cryophila* PKS proteins. Figure S21: Structure prediction for macrolactone core of *Cross. cryophila* polyene. Figure S22: Domain compositions of *Amyc. antarctica* PKS protein. Figure S23: Domain compositions of *Amyc. algeriensis* PKS proteins. Figure S24: Structure prediction for macrolactone core of *Amyc. algeriensis* polyene. Table S1: Genome accession numbers and isolation source for microorganisms containing cryptic polyene biosynthetic gene clusters. Table S2: Mycosamine transferases and polyene extending GTs. Supplementary Excel File S1: Tables of genes from cryptic polyene BGCs. Supplementary Excel File S2: Motifs used for stereostructure prediction.
