# Supplementary material for: New Glycosylated Polyene Macrolides: Refining the Ore from Genome Mining
Source: Antibiotics (Basel). 2022 Mar 3;11(3):334. doi: 10.3390/antibiotics11030334 (PMC8944477; doi:10.3390/antibiotics11030334)
Supplement: Supplementary file 1 [file antibiotics-11-00334-s001.zip › antibiotics-1597859-Supplementary tables and figures.pdf]

New glycosylated polyene macrolides: refining the ore from genome mining

Patrick Caffrey, Mark Hogan, and Yuhao Song

Supplementary Information

Table S1. Genome accession numbers and isolation source for micro-organisms containing cryptic polyene biosynthetic gene clusters.

Fig. S1 Domain compositions of *Amyc. saalfeldensis* PKS proteins
Fig. S2 Schematic diagram of final polyketide chain made by *Amyc. saalfeldensis* PKS
Fig. S3 Structure prediction for macrolactone core of *Amyc. saalfeldensis* polyene
Fig. S4 Domain compositions of *Amyc. albispora* PKS proteins
Fig. S5 Structure prediction for macrolactone core of *Amyc. albispora* polyene
Fig. S6 Domain compositions of *Sacc. dendrathemae* PKS proteins
Fig. S7 Structure prediction for macrolactone core of *Sacc. dendrathemae* polyene
Fig. S8 Domain compositions of *Amyc. lexingtonensis* PKS proteins
Fig. S9 Structure prediction for macrolactone core of *Amyc. lexingtonensis* polyene

Table S2. Protein accession numbers for extending GTs and paired mycosaminyltransferases

Fig. S10 Alignment of polyene extending GTs and mycosaminyltransferases
Fig. S11 Alignment of PegA and putative extending GTs from *Crypto. arvum* and *Amyc. suaedae*

Fig. S12 Domain compositions of *Crypto. arvum* PKS proteins
Fig. S13 Structure prediction for macrolactone core of *Crypto. arvum* polyene
Fig. S14 Domain compositions of *Amyc. suaedae* PKS proteins
Fig. S15 Structure prediction for macrolactone core of *Amyc. suaedae* polyene
Fig. S16 Domain compositions of *Sacc. gloriosae* PKS proteins
Fig. S17 Structure prediction for macrolactone core of *Sacc. gloriosae* polyene
Fig. S18 Domain compositions of *Amyc. cihanbeyliensis* PKS proteins
Fig. S19 Structure prediction for macrolactone core of *Amyc. cihanbeyliensis* polyene
Fig. S20 Domain compositions of *Cross. cryophila* PKS proteins
Fig. S21 Structure prediction for macrolactone core of *Cross. cryophila* polyene
Fig. S22 Domain compositions of *Amyc. antarctica* PKS protein
Fig. S23 Domain compositions of *Amyc. algeriensis* PKS proteins
Fig. S24 Structure prediction for macrolactone core of *Amyc. algeriensis* polyene

Supplementary Excel File S1. Tables of genes from cryptic polyene BGCs

Supplementary Excel File S2. Motifs used for stereostructure prediction

Table S1. Genome accession numbers and isolation sources for micro-organisms containing cryptic BGCs

**Microorganism**	**Genome accession**	**Isolation source**
Acrocarpospora macrocephala	NZ_BLAE01000020.1	Soil sample, Saitama prefecture, Japan
Acrocarpospora pleiomorpha	NZ_BLAF01000005.1	Soil, Louisiana, USA
Actinokineospora mzabensis	NZ_QHCP01000004	Saharan soil, Southern Algeria
Actinokineospora spheciospongiae DSM45935	GCA_000564855.1	Red Sea sponge, Egypt
Actinophytocola algeriensis	JACHJQ010000010	Saharan soil sample Southern Algeria
Actinophytocola xanthii	MSIE01000019.1	*Xanthium sibiricum* plant, China
Actinoplanes digitatis DSM43149	JACHNH010000001	Soil sample, origin not recorded
Amycolatopsis albispora	CP015163.1	Deep-sea sediment, Indian Ocean
Amycolatopsis antarctica	NZ_NKYE01000003	Brown macroalga, the Antarctic
Amycolatopsis benzoatilytica	NZ_KB912942	Czech patient with sub-mandibular mycetoma
Amycolatopsis cihanbeyliensis	NZ_VFML01000001, NZ_VFML01000002	Cihanbeyli salt mine, Turkey
Amycolatopsis eburnei	NZ_RSEC01000046.1	Arbuscular mycorrhizal fungal spores
Amycolatopsis jejuensis	NZ_JNYZ01000006	Cave on the island of Jeju, Korea
Amycolatopsis lexingtonensis	JADBEG010000001	Lesions on equine placenta, Lexington, USA
Amycolatopsis saalfeldensis	FOEF01000000	Medieval alum slate mine, Saalfeld, Germany
Amycolatopsis suaedae	NZ_SFCC01000005, NZ_SFCC01000018	*Suaeda maritima* salt marsh plant, Thailand
Amycolatopsis YIM10	CP045480.1	Rare earth mine, Inner Mongolia, China
Couchioplanes caeruleus	NZ_RJKL01000001	Soil sample, California, USA
Crosiella cryophila DSM44230	JACHMH010000001.1	Soil sample, Japan

Cryptosporangium arvum DSM44712	JFBT01000001	Soil sample, Japan
Lentzea waywayandensis	NZ_FOYL01000001.1	Soil, Lake Waywayanda, New Jersey, USA.
Lentzea xinjiangensis	NZ_FOFR01000005	Soil sample, Xinjiang in China.
Pseudonocardia endophytica	NZ_SMFZ01000001.1	Endophyte of *Lobelia clavata* plant, China
Saccharopolyspora dendrathemae DSM46699	NZ_VIWX01000004.1	Endophyte of a coastal salt marsh plant, China
Saccharopolyspora flava	NZ_FOZX01000003	Garden soil sample, China
Saccharopolyspora gloriosae	JACHIV010000001	Endophyte of *Gloriosa superba* plant, China
Streptomyces bingchengensis	CP002047.1	Soil sample, Harbin, China
Streptomyces eurocidicus	NZ_LGUI101000004; NZ_LGUI01000000	Soil sample, origin not recorded
Streptomyces milbemycinus DSM41911	NZ_MUNE01000199.1; NZ_MUNE01000198.1	Soil sample, Indiana, USA
Streptomyces noursei	CP011533.1	Soil sample, Fauquier County, Virginia, USA
Streptomyces 14.10	NZ_NCSM01000015.1; NZ_NCSM01000025.1	River sediment in Brazil
Streptosporangium album DSM43023	JACHJU010000004.1; JACHJU010000008.1	Soil sample, Japan

PKS1 (WP_218156697.1)
ACP_L-KS1-AT1-DH1-ER1-$KR1_B$-ACP1-KS2-AT2-$KR2_B$-ACP2-KS3-AT3-DH3-$KR3_B$-ACP3

PKS2 (WP_091613189.1)
KS4-AT4-DH4-ER4-$KR4_B$-ACP4-KS5-$AT5_{MM}$-$KR5_{B1}$-ACP5

PKS3 (WP_091614516.1)
KS6-$AT6_{MM}$-$DH6^0$-$KR6_{B1}$-ACP6

PKS4 (WP_091613192.1)
KS7-AT7-DH7-$KR7_B$-ACP7-KS8-AT8-DH8-$KR8_B$-ACP8-KS9-AT9-DH9-$KR9_B$-ACP9-KS10-AT10-DH10-$KR10_B$-ACP10

PKS5 (WP_091613167.1)
KS11-AT11-DH11-$KR11_B$-ACP11-KS12-AT12-$KR12_A$-ACP12-KS13-$AT13_{MM}$-$KR13_{A2}$-ACP13-KS14-AT14-$KR14_B$-ACP14-KS15-AT15-$KR15_0$-ACP15-KS16-AT16-$KR16_B$-ACP16

PKS6 (WP_091613170.1)
KS17-AT17-$KR17_B$-ACP17-KS18-AT18-DH18-$KR18_B$-ACP18

PKS7 (WP_091613172.1)
KS19-$AT19_{MM}$-DH19-$ER19_S$-$KR19_{B1}$-ACP19-TE

Figure S1. Domain compositions of *Amyc. saalfeldensis* PKS proteins. Seven PKS proteins were identified. Accession numbers for protein sequences are given. AT domains specific for methylmalonyl CoA are designated ATX_{MM} e. g. $AT5_{MM}$, all other AT domains are malonyl CoA specific. KR domain subgroups are designated as subscripts e. g. $KR1_B$, $KR5_{B1}$, $KR13_{A2}$ etc. Inactive DH domains are designated with zero as a superscript e. g. $DH6^0$. The subscript S after an ER domain indicates that it gives a 2*S*-2-methylbranched polyketide intermediate, e. g. $ER19_S$. The genes are listed in Excel file S1 sheet A. The motifs important for structure prediction are shown in Excel file S2 sheet 1.

Guanidinobutyryl CoA

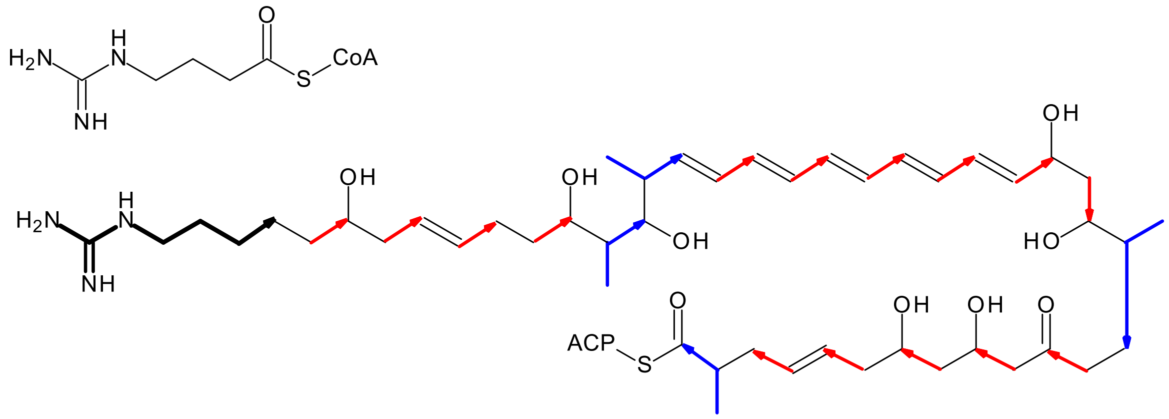

Figure S2. Schematic representation of final polyketide intermediate produced by *Amyc. saalfeldensis* PKS. The chain is drawn as an acyl thioester on ACP19 and folded so as to favour hemiketal formation and cyclisation. The black arrow represents the guanidinobutyryl primer, red arrows represent malonyl-derived acetyl extender units, and blue arrows represent methylmalonyl-derived propionyl extender units.

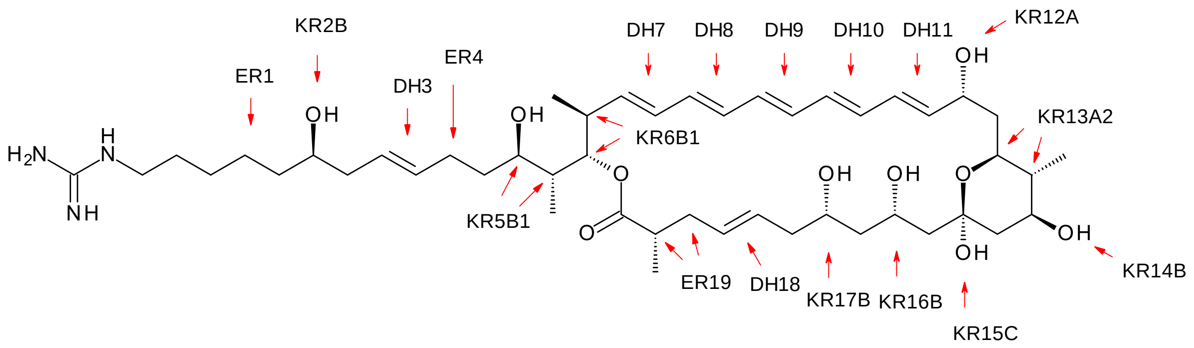

Figure S3. Structure prediction for the *Amyc. saalfeldensis* polyketide. The domains that form the various functional groups derived from β-ketones are indicated. The stereochemistry imposed by KR domains or ER19 is shown. All DH domains are paired with B-type KRs, so all double bonds are predicted to have *trans* geometry.

PKS 1 (AXB47627.1)
CoAL-KR$_C$-ACP-KS1-AT1-KR1$_A$-ACP

PKS 2 (AXB47628.1)
KS2-AT2$_{MM}$-DH2-KR2$_B$-ACP2-KS3-AT3-DH3-KR3$_B$-ACP3-KS4-AT4-DH4-KR4$_B$-ACP4-KS5-AT5$_{MM}$-DH5-KR5$_{B1}$-ACP5-KS6-AT6$_{EM}$-KR6$_{A1}$-ACP6-KS7-AT7-KR7$_B$-ACP7-KS8-AT8$_{EM}$-KR8$_{A1}$-ACP8

PKS 3 (AXB47629.1)
KS9-AT9-DH9-KR9$_B$-ACP9- KS10-AT10-DH10-KR10$_B$-ACP10-KS11-AT11-DH11-KR11$_B$-ACP11-KS12-AT12-DH12-KR12$_B$-ACP12-KS13-AT13-DH13-KR13$_B$-ACP13-KS14-AT14-DH14-KR14$_B$-ACP14

PKS 4 (AXB47619.1)
KS15-AT15-DH15-KR15$_B$-ACP15-KS16-AT16-KR16$_A$-ACP16-KS17-AT17$_{MM}$-KR17$_{A2}$-ACP17-KS18-AT18-KR18$_B$-ACP18-KS19-AT19-ACP19-KS20-AT20-KR20$_B$-ACP20

PKS 5 (AXB47620.1)
KS21-AT21-DH21-ER21-KR21$_B$-ACP21-KS22-AT22-DH22-ER22-KR22$_B$-ACP22-KS23-AT23-KR23$_B$-ACP23-KS24-AT24-DH24^0-KR24$_B$-ACP24

PKS 6 (AXB47621.1)
KS25-AT25-KR25$_B$-ACP25-TE

Figure S4. Domain compositions of *Amyc. albispora* PKS proteins. AntiSMASH predicts that the CoA ligase in the starter unit is specific for 3-amino-5-hydroxy-benzoic acid [58]. Domains are labelled as in Figure S3. AT6$_{EM}$ and AT8$_{EM}$ are predicted to be ethylmalonate-specific. The genes in the polyene BGC are listed in Excel file 1 sheet B. The motifs relevant for structure prediction are listed in Excel file 2 sheet 2.

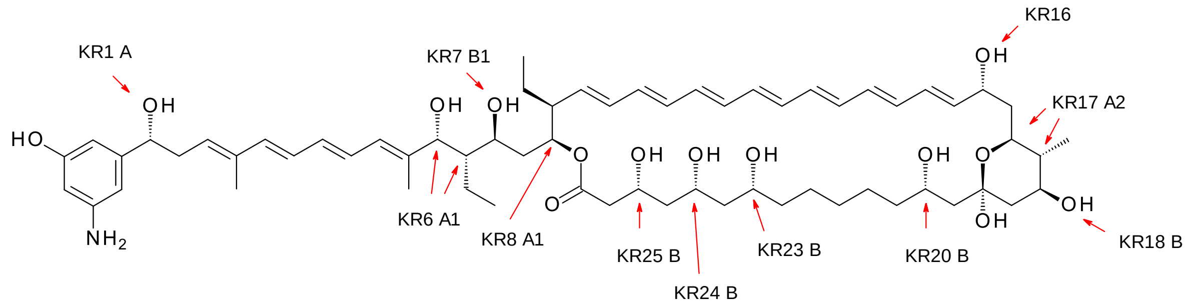

Figure S5. Structure prediction for the *Amyc. albispora* polyketide. All DH domains are paired with B-type KRs, so all double bonds are predicted to have *trans* geometry.

PKS1 (WP_186459512.1)
CAL-KR0$_C$-ACP0-KS1-AT1-KR1$_B$-ACP1

PKS2 (WP_145741813.1)
KS2-AT2-DH2-KR2$_B$-ACP2- KS3-AT3-DH3-KR3$_B$-ACP3

PKS3 (WP_145741810.1)
KS4-AT4-DH4-KR4$_A$-ACP4- KS5-AT5$_{MM}$-KR5$_{A1}$-ACP5- KS6-AT6-KR6$_B$-ACP6-KS7-AT7$_{EM}$-KR7$_{A1}$-ACP7

PKS4 (WP_145741808.1)
KS8-AT8-DH8-KR8$_B$-ACP8-KS9-AT9-DH9-KR9$_B$-ACP9-KS10-AT10-DH10-KR10$_B$-ACP10-KS11-AT11-DH11-KR11$_B$-ACP11-KS12-AT12-DH12-KR12$_B$-ACP12-KS13-AT13-DH13-KR13$_B$-ACP13

PKS5 (WP_145742606.1)
KS14-AT14-DH14-KR14$_B$-ACP14- KS15-AT15-KR15$_A$-ACP15- KS16-AT16$_{MM}$-KR16$_{A2}$-ACP16-KS17-AT17-KR17$_B$-ACP17-KS18-AT18-KR18$_C$-ACP18-KS19-AT19-KR19$_B$-ACP19

PKS6 (WP_145741839.1)
KS20-AT20-DH20^0-KR20$_B$-ACP20-KS21-AT21-KR21$_C$-ACP21-KS22-AT22-DH22-ER22-KR22$_B$-ACP22-KS23-AT23-DH23^0-KR23$_B$-ACP23

PKS7 (WP_145741837.1)
KS24-AT24-KR24$_B$-ACP24-TE

Figure S6. Domain compositions of *Sacc. dendranthemae* PKS proteins. Domains are labelled as in figures S1 and S4. The genes in the polyene BGC are listed in Excel file 1 sheet C. The motifs relevant for structure prediction are listed in Excel file 2 sheet 3.

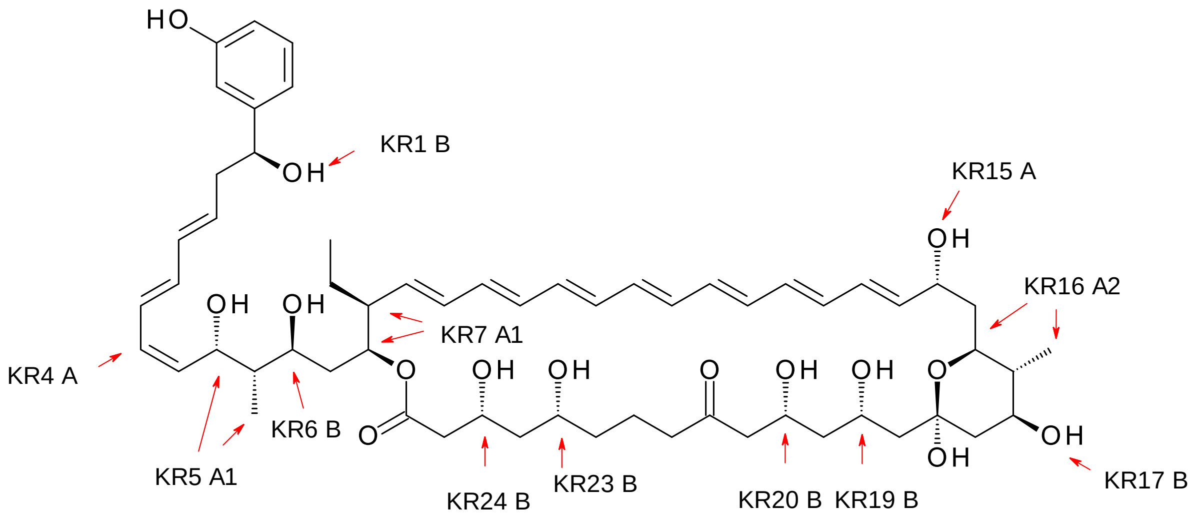

Figure S7. Structure prediction for the *Sacc. dendrathemae* polyketide. All DH domains are paired with B-type KRs, so all double bonds are predicted to have *trans* geometry.

PKS1 (MBE1496553.1)
KS^{Q}-AT$_{L}$-ACP$_{L}$-KS1-AT1-DH1-KR1$_{B}$-ACP1-KS2-AT2-DH2-KR2$_{B}$-ACP2-KS3-AT3-DH3-KR3$_{B}$-ACP3-KS4-AT4-DH4-KR4$_{B}$-ACP4-KS5-AT5-KR5$_{B}$-ACP5

PKS2 (MBE1496564.1)
KS6-AT6-DH6-KR6$_{B}$-ACP6-KS7-AT7-KR7$_{A}$-ACP7-KS8-AT8$_{MM}$-KR8$_{A2}$-ACP8-KS9-AT9-KR9$_{B}$-ACP9-KS10-AT10-KR10$_{C}$-ACP10-KS11-AT11-KR11$_{B}$-ACP11

PKS3 (MBE1496563.1)
KS12-AT12-DH12^{0}-KR12$_{B}$-ACP12-KS13-AT13-DH13-ER13-KR13$_{B}$-ACP13-KS14-AT14-KR14$_{B}$-ACP14-KS15-AT15-DH15-KR15$_{B}$-ACP15-KS16-AT16-DH16-KR16$_{B}$-ACP16

PKS4 (MBE1496562.1)
KS17-AT17$_{MM}$-ACP17-KS18-AT18-DH18-KR18$_{B}$-ACP18

PKS5 (MBE1496558.1)
KS19-AT19-DH19^{0}-ACP19-KS20-AT20-KR20$_{B}$-ACP20

PKS6 (MBE1496557.1)
KS21-AT21-DH21-KR21$_{B}$-ACP21-KS22-AT22-DH22-KR22$_{B}$-ACP22-KS23-AT23-DH23-KR23$_{B}$-ACP23-KS24-AT24$_{MX}$-KR24$_{B}$-ACP24

PKS7 (MBE1496556.1)
KS25-AT25-DH25-KR25$_{B}$-ACP25-KS26-AT26-KR26$_{B}$-ACP26-KS27-AT27-DH27-KR27$_{B}$-ACP27-KS28-AT28-DH28-KR28$_{B}$-ACP28

PKS (MBE1496555.1)
KS29-AT29$_{MM}$-DH29-KR29$_{B}$-ACP29-TE

Figure S8. Domain compositions of *Amyc. lexingtonensis* PKS proteins. AT24$_{MX}$ is predicted to be specific for methoxymalonate. The *Amycolatopsis lexingtonensis* genome has accession number JADBEG010000001. The genes in the polyene BGC are listed in Excel file 1 sheet D. The motifs relevant for structure prediction are listed in Excel file 2 sheet 4.

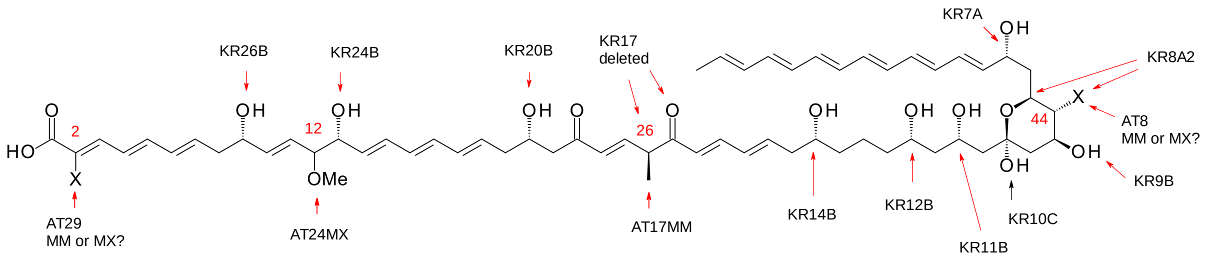

Figure S9. Stereostructure predicted for *Amyc. lexingtonensis* hexaene.

Micro-organism	Mycosamine transferase Accession	Short name	Extending GT Accession	Short name	Ref
Ps. autotrophica KCTC9441	WP_073575130.1	MTr1	WP_083658368.1	EGT1 (GlcNAc)	[82]
Ps. autotrophica DSM4308	"	"	"	"	[91]
Ps. SID8383	"	"	"	"	[92]
Ps. antarctica DSM44749	"	"	WP_179760056.1	EGT1A (GlcNAc)	[94]
Ps. alni DSM44104	WP_100877978.1	MTr2	WP_100877988.1	EGT2 (GlcNAc)	[93]
Ps. AL041005-10	WP_062394421.1	MTr3	WP_062394400.1	EGT3 (GlcNAc)	[95]
Ps. P1	WP_010232932.1	MTr4	WP_010243778.1	EGT4 (Mannose)	[81]
Ps. Ae707P-1	"	"	"	"	[66]
Ps. ECO80610-09	WP_060575838.1	MTr5	WP_06057583.1	EGT5 (Mannose)	[96]
Ps. ECO80619-01	"	"	"	"	[96]
Ps. Ae150-Ps1	WP-075294936.1	MTr6	WP_075295677.1	EGT6 (Mannose)	[66]
Ps. Ae168-Ps1	"	"	"	"	[66]
Ps. Ae263-Ps1	"	"	"	"	[66]
Ps. Ae356-Ps1	"	"	"	"	[66]
					[66]
Couchioplanes caeruleus	WP_071802619.1	MTr7	WP_071803650.1	EGT7 (Mannose)	[79]
Crypto. arvum DSM 44712	EXG82139.1	MTr8	EXG82143.1	EGT8	[97]
Amyc. suaedae	WP_130478878	MTr9	WP_130478880.1	EGT9	[99]

Table S2. Mycosamine transferases and polyene extending GTs.

```
MTr7        -----------------MNTDPRAVMFVSLPESGLLNPMLVLAAELARRGVPGLSFATDE    43
MTr6        -----------------MGANRRPILFVSYAESGLLNPLLVLAGELSRREVGDLFFAADE    43
MTr4        -----------------MGANRRPILFVSYAESGLLNPLLVLAGELSRREVGDLFFAADE    43
MTr5        -----------------MGANRRPILFVSYAESGLLNPLLVLAGELSRREVGDLFFAADE    43
MTr3        -----------------MGANRRPILFVSYAESGLLNPLLVLAGELSRREVADLYFAADE    43
MTr1        -----------------MGANRRPILFVSYAESGLLNPLLVLAGELSRREVGDLYFAADE    43
MTr2        -----------------MGANRRPILFVSYAESGLLNPLLVLAGELSRREVGDLYFAADE    43
EGT7        ------------------MTSHRPILFCCTHSTGQATSSLVLAGEFAARGVKNLWFASDD    42
EGT6        MEQTTGTRPADDAAQQADGAGAQPILFCCTHSTGEAATSLVLAGELARRGVPDLVFAADE    60
EGT4        MEQTTGTRPADDAAQQADGAGAQPILFCCTHSTGEAATSLVLAGELARRGVPDLVFAADE    60
EGT5        MEQTTGTRPADDAAQQADGAGAQPILFCCTHSTGEAATSLVLAGELARRGVPDLVFAADE    60
EGT3        MEQTTGTQRADGPGQEADQAGARPVLFCCTHSTGEAATSLALAGELARRGVPDLVFAADE    60
EGT2        MEQTTGTQRADGPGQEADQAGARPVLFCCTHSTGEAATSLALAGELARRGVPDLVFAADE    60
EGT1        MEQTTGTQRADGPGQEADQAGARPVLFCCTHSTGEAATSLALAGELARRGVPDLVFAADE    60
EGT1A       MEQTTGTQRADGPGQEADQAGARPVLFCCTHSTGEAATSLALAGELARRGVPDLVFAADE    60
                               :. : ::* .  .:*     *.**.*:: * * .* **:*:

MTr7        SRRTDIETISGDHPVRFASLGEVVPELSAVTWDDATYRRVTQRSRFKAHRAVIRHTYRPD    103
MTr6        KARDDVESAGTGSPVTFTSLGDTVSEMSAVTWDDETYAAVTQRDRFKAHAAVIRHSFAPE    103
MTr4        KARDDVESAGTGSPVTFTSLGDTVSEMSAVTWDDETYAAVTQRDRFKAHAAVIRHSFAPE    103
MTr5        KARDDVESAGTGSPVTFTSLGDTVSEMSAVTWDDETYAAVTQRDRFKAHAAVIRHSFAPE    103
MTr3        KARDDVAAAGTGSPVTFTSLGDTVSEMSAVTWDDETYAAVTQRDRFRAHAAVIRHSFAPE    103
MTr1        KARDDVAGASTGSPVTFASLGDTVSEMSAVTWDDETYAAVTQRDRFRAHAAVIRHSFAPE    103
MTr2        KARDDVAGASTGSPVTFASLGDTVSEMSAVTWDDETYAAVTQRDRFKAHAAVIRHSFAPE    103
EGT7        NHRAAVAGLSGASEVGFVSTGPVNPRVAPTMWDDETYRSITQRSRWKGNRARLRQLMDPV    102
EGT6        NLRGPVGELADRSAVEFVSLGPVNPDLALTMIDDATYARIHQRSRVRGLRARARQLFDVD    120
EGT4        NLRGPVGELADRSAVEFVSLGPVNPDLALTMIDDATYARIHQRSRVRGLRARARQLFDVD    120
EGT5        NLRGPVGELADRSAVEFVSLGPVNPDLALTMIDDATYARIHQRSRVRGLRARARQLFDVD    120
EGT3        NLRGPVEELADRSAVEFVSLGPVNPALALTMIDDATYARIHQRSRVRGLRARARQLFDVD    120
EGT2        NLRGPVEELADRSAVEFVSLGPVNPALALTMMDDATYDRIYQRSRVRALRARARQLFDVD    120
EGT1        NLRGPVEEIADRSAVEFVSLGPVNPALALTMMDDATYDRIYQRSRVRALRARARQLFDVD    120
EGT1A       NLRGPVEELADRSAVEFVSLGPVNPALALTMMDDATYDRIYQRSRVRALRARARQLFDVD    120
            . *  :   .    * *.* * .   :: .  ** **  : **.* :.  *  *:

MTr7        LQATKFRALAAAVDEIQPELMVIESLCSFAVDLALTRGIPFVLSVPFLPSNVLTAHNPFA    163
MTr6        SRIEKYRALERVVEEARPALMVIESMCQYGYELAITKGIPFVLGVPFVPSNVLTSHVPFA    163
MTr4        SRIEKYRALERVVEEVRPALMVIESMCQYGYELAITKGIPFVLGVPFVPSNVLTSHVPFA    163
MTr5        SRIEKYRALERVVEEVRPALMVIESMCQYGYELAITKGIPFVLGVPFVPSNVLTSHVPFA    163
MTr3        SRIEKYRALERVVDEVRPALMVIESMCQYGYELAITQGIPFVLGVPFVPSNVLTSHVPFA    163
MTr1        SRIEKYRALERVVDEVRPALMVIESMCQYGYELAITKGIPFVLGVPFVPSNVLTSHVPFA    163
MTr2        SRIEKYRALERVVDEVRPALMVIESMCQYGYELAITKGIPFVLGVPFVPSNVLTSHVPFA    163
EGT7        HHADRFRQLDAAVQRIQPALMVINNLCIHGIQVAMKRGVPYVITGSSLASDIWQF-----    157
EGT6        HLMQRYQALDEVVERVRPALMVINRFATHAVQVALTRGIPYVITAPCLLSSLVEH-----    175
EGT4        HLMQRYQALDEVVERVRPALMVINRFATHAVQVALTRGIPYVITAPCLLSSLVEH-----    175
EGT5        HLMQRYQALDEVVERVRPALMVINRFATHAVQVALTRGIPYVITAPCLLSSLVEH-----    175
EGT3        HLVQRYQALDEVVERVRPALMVINRFATHALQVALTRGIPYVITAPCLLSSLIEH-----    175
EGT2        HLLQRYQALDEVVERVRPALMVINRFATHAVLVALARGIPYVITAPCLLSSLVEH-----    175
EGT1        HLLQRYQALDEVVERVRPALMVINRFATHAVLVALARDIPYVITAPCLLSSLVEH-----    175
EGT1A       HLLQRYQALDEVVERVRPALMVINRFATHAVLVALARGIPYVITAPCLLSSLVEH-----    175
                ::: *  .*:. :* ****: :. ..  :*: :.:*:*:    : *.:

MTr7        RSYTPRGFPVPHSGLPARMTPWQRLSNEMFKLRTLAMFFHPAMGRVLSEDARIRKELGLS    223
MTr6        TSYTPSGFPVPHSGLPADMTLRQKVANQLFRIRTLGMFVTKETRKVVERDDAVRAELGIA    223
MTr4        TSYTPSGFPVPHSGLPADMTLRQKITNQLFRIRTLGMFVTKETRKVVERDDAVRAELGIA    223
MTr5        TSYTPSGFPVPHSGLPADMTLRQRITNQLFRIRTLGMFVTKETRKVVERDDAVRAELGIA    223
MTr3        RSYTPSGFPVPHSGLPATMTLRQKIANQLFRIRTLGMFVTKETRKVVERDDAVRAELGIA    223
MTr1        RSYTPSGFPVPHSGLPATMTLRQKIANQLFRIRTLGMFVTKETRKVVERDDAVRAELGIA    223
MTr2        RSYTPSGFPVPHSGLPAAMTLRQKIANQLFRIRTLGMFVTKETRAVVERDDAVRAELGIA    223
EGT7        --DLPDDYPVPYSGLPRRMNLRQRIGNRLFRHRNLLSMLDPALLRTVGAMLKATDELGLD    215
EGT6        --DLPRGFPPPSSGLPLHRTRRQELERIWFGIGTGTLFLDRSVFRKAVRLHRGMGELGID    233
EGT4        --DLPRGFPPPSSGLPLHRTRRQELERIWFGIGTGTLFLDRSVFRKAVRLHRGMGELGID    233
EGT5        --DLPRGFPPPSSGLPLHRTRRQELERIWLGIGTGTLFLDRSVFRKAVRLHRGMGELGID    233
EGT3        --DLPRGFPPPSSGLPLRRTWRQELERIWFTVGTGTVFLDRAVLRSAVRLHRRMGELGID    233
EGT2        --DLPRGFPPPSSGLPLRRTLRQELDRIWFRIGVGSLFLDRSVLRRAVRLHKRMGELGLD    233
EGT1        --DLPRGFPPPSSGLPLRRTLRQELDRIWFRIGVGSLFLDRSVLRRAVRLHKRMGELGLD    233
EGT1A       --DLPRGFPPPSSGLPLRRTLRQELDRIWFRIGVGSLFLDRSVLRRAVRLHKRMGELGLD    233
                * .:* * ****   .  *.: .  :       :.                ***:
```

```
MTr7        V--PSPMTRVDRAEMVICNSIPELDYPFDIPDKLKLVGALMPPLPEASTDNEVMRWLAAQ    281
MTr6        PEARQMMARIDHAEQVLCYSVPELDYPFDLHEKLRLVGTMVPPLPQVADGGELTDWLDEN    283
MTr4        PEARQMMARIDHAEQVLCYSVPELDYPFDLHEKLRLVGTMVPPLPQVADGGELTDWLDEN    283
MTr5        PEARQMMARIDHAEQVLCYSVPELDYPFDLHEKLRLVGTMVPPLPQVADGGELTDWLDEN    283
MTr3        PEARQMMARIDHAEQVLCYSVPELDYPFELPEKMRLVGTMVPPLPQVADGGELTEWLDAQ    283
MTr1        PEARQMMARIDHAEQVLCYSVPELDYPFELPERMRLVGTMVPPLPQVADGGELTDWLDAQ    283
MTr2        PEARQMMARIDHAEQVLCYSVPELDYPFELPERMRLVGTMVPPLPQVADGGELTDWLDAQ    283
EGT7        RRQLQQRAWMEEAELVLCFSVFGLDYPFPHPPKLQMVGAMVPPLPQNPVPGETDRWLDAH    275
EGT6        PRTLRVPVQNAGARSLLCFSVPGVDYPLPVPDRVRMVGALVPPVRHDERDAGVAEWLDAH    293
EGT4        PRTLRVPVQNAGARSMLCFSVPGVDYPLPVPDRVRMVGALVPPVRHDERDAGVAEWLDAH    293
EGT5        PRTLRVPVQNAGARSMLCFSVPGVDYPLPVPDRVRMVGALVPPVRHDERDAGVAEWLDAH    293
EGT3        PRTLRAAVHNAGARSVLLFSVPGVDYPLAAPERVRMVGALVPPDRHDERDAEVTEWLDAH    293
EGT2        PRTLRAPVQQEGARSLLCFTVPGVDYPLPVPDRVRMVGALVPPSRHDERDAAVTEWLDAH    293
EGT1        PRTLRAPVQQEGARSLLCFTVPGVDYPLPVPDRVRMVGALVPPSRHDERDAAVTEWLDAH    293
EGT1A       PRTLRAPVQQEGARSLLCFTVPGVDYPLPVPDRVRMVGALVPPSRHDERDAAVTEWLDAH    293
                   .    *. ::  ::  :***:    ::::**:::**  .         **  :

MTr7        RSVVYMGFGTITRLSTAQAGALVEVARRLDG-THSVLWRLPSEQQHLLADAGPLPANLRI    340
MTr6        PSVVYMGFGTITRLTRPQVASLVEVARRLDEQGHQVLWKLPREQQEMLPPADELPGNLRI    343
MTr4        PSVVYMGFGTITRLTRPQVASLVEVARRLDEQGHQVLWKLPREQQEMLPPADELPGNLRI    343
MTr5        PSVVYMGFGTITRLTRPQVASLVEVARRLDEQGHQVLWKLPREQQEMLPPADELPGNLRI    343
MTr3        SSVVYMGFGTITRLTRPQVASLVEVARRLDARGHQVLWKLPREQQEMLPPAAELPAGLRI    343
MTr1        SSVVYMGFGTITRLTRPQVASLVEVARRLDARGHQVLWKLPREQQEMLPPATELPAGLRI    343
MTr2        SSVVYMGFGTITRLTRPQVASLVEVARRLDARGHQVLWKLPREQQEMLPPATELPAGLRI    343
EGT7        PSVVFIAFGTITRLTKAEVRAVVDVARRLGE-RHHVLWKLPREQQRFLPPAAELPANLRV    334
EGT6        PSTVYLAFGSITRMTADQIRSVVELAHRLGD-DHGILWVLRADQQRFLPDPADRPANLKV    352
EGT4        PSTVYLAFGSITRMTADQVRSVVELARRLGD-DHGVLWVLRADQQRFLPDPADRPANLKV    352
EGT5        PSTVYLAFGSITRMTADQVRSVVELARRLGD-DHGVLWVLRADQQRFLPDPADRPANLKV    352
EGT3        PSVVYTAFGSITRMTPDQVGSVVDLARRLGD-DHGLLWVLHRDQQRFLP--ADLPPNLKV    350
EGT2        PSVVYVAFGSVTRMTADQVRSLVELARRLGD-DHGLLWVLHRDQQRLLP--AELPANLKV    350
EGT1        PSVVYVAFGSVTRMTADQVRSLVELARRLGD-DHGLLWVLHRDQQRLLP--AELPANLKV    350
EGT1A       PSVVYVAFGSVTRMTADQVRSLVELARRLGD-DHGLLWVVHRDQQRLLP--AELPANLKV    350
             *.*: .**::**::  :  ::*::*:**.   * :** :  :**.:*      * .*::

MTr7        ENWLPSQLDVLAHPAVKVFFTHGGGNGFHEGVYFGKPLVVRPLWVDCFDQAIRGRDAGVS    400
MTr6        ESWVPSQLDVLAHPSVKVFFTHAGGNGYHEGLYFGKPLVVRPLWVDCDDQAVRGQDFGVS    403
MTr4        ESWVPSQLDVLAHPSVKVFFTHAGGNGYHEGLYFGKPLVVRPLWVDCDDQAVRGQDFGVS    403
MTr5        ESWVPSQLDVLAHPSVKVFFTHAGGNGYHEGLYFGKPLVVRPLWVDCDDQAVRGQDFGVS    403
MTr3        ESWVPSQLDVLAHPGVKVFFTHAGGNGYHEGLYFGKPLVVRPLWVDCDDQAIRGQDFGVS    403
MTr1        ESWVPSQLDVLAHPSVKVFFTHAGGNGYHEGLYFGKPLVVRPLWVDCDDQAIRGQDFGVS    403
MTr2        ESWVPSQLDVLAHPSVKVFFTHAGGNGYHEGLYFGKPLVVRPLWVDCDDQAIRGQDFGVS    403
EGT7        EDWLPSQYDVLAHSNVRVFFGHGGGNNSFHEGIYFGKPSLVRPLWFDCLDHAVRAVDSGVG    394
EGT6        VDWLHSQHAVLEHPHVRAFFTHGGSNSIHESLWFGTPVLVRPTNVDQYDHAVRATDTGIG    412
EGT4        VDWLHSQHAVLEHPHVRAFFTHGGSNSIHESLWFGTPVLVRPTNVDQYDHAVRATDTGIG    412
EGT5        VDWLHSQHAVLEHPHVRAFFTHGGSNSIHESLWFGTPVLVRPTNVDQYDHAVRATDTGIG    412
EGT3        VPWVHSQLGVLEHPHVRVFFTHGGSNSVHESLYFGVPVLVRPTNVDQYDHAVRAVDTGIG    410
EGT2        VPWVHSQLGVLEHPHVRAFFTHGGSNSIHESLYFGVPVLVRPTLVDQFDHAVRAVDTGIG    410
EGT1        VPWVHSQLGVLEHPHVRAFFTHGGSNSIHESLYFGVPVLVRPTLVDQFDHAVRAVDTGIG    410
EGT1A       VPWVHSQLGVLEHPHVRAFFTHGGSNSIHESLYFGVPVLVRPTLVDQFDHAVRAVDTGIG    410
              *: **  ** *  *:.** *.*.*. **.::** * :***  .*  *:*:*. * *:.
```

```
MTr7            LTLDHPETVDPDDVLDKLNRVLDDPSFTARAAQLGARQREAGGVRAAADLINQVVTR---     457
MTr6            LTVDHPETVDTADVLDKVTRVLDDPAFTERAAHMGRLLQEAGGRAAAADLLLGLPALAGS     463
MTr4            LTVDHPETVDTADVLDKVTRVLDDPAFTERAAHMGRLLREAGGRTAAADLLLGLPALAGS     463
MTr5            LTVDHPETVDTADVLDKVTRVLDDPAFTERAAHMGRLLQEAGGRAAAADLLLGLPALAGS     463
MTr3            LTVDHPETVDTEDVLDKVTRVLETPAFTERAERMGRLLQAAGGRVAAADLVLRLPALAGT     463
MTr1            LTVDHPETVDTDDVLDKVTRVLETPAFTERAARMGRLLHEAGGRAAAADLILRLPVLAGT     463
MTr2            LTVDHPETVDTEDVLDKVTRVLETPAFTERAERMGRLLQAAGGRAAAADLVLRLPALAGT     463
EGT7            LSVA-PGTMDPAEIHSKLTALLDDTSFRARAEHFAQIQHEAGGVRRAADLILRCAAVTSA     453
EGT6            LAVDRPDLVDVDDVHAKLQRLLREPAFTERARELGDVQRAAGGLDTAADAVLAELGNRHP     472
EGT4            LAVDRPDLVDVDDVHAKLQRLLREPAFTERARELGDVQRAAGGLDTAADAVLAELGNRHP     472
EGT5            LAVDRPDLVDVDDVHAKLQRLLREPAFTERARELGDVQRAAGGLDTAADAVLAELGNRHP     472
EGT3            LAVERPDRIDVEDTHDRLLQLLHEPGFADRAREIGRVQRSAGGLRVAGDAVLTELREASV     470
EGT2            LTVERPDRIDVDDTRDRLLRLLHEPGFADRAREIGQVQRSAGGLRVAGDAVLMELREASV     470
EGT1            LTVERPDRIDVDDTHGRLLRLLHEPGFADRAREIGQVQRSAGGLRVAGDAVLMELREASV     470
EGT1A           LTVERPDRIDVDDTHGRLLRLLHEPGFADRAREIGQVQRSAGGLRVAGDAVLMELREASV     470
                *::  *  :*  :   ::  :*   .*  ** .:.   : ***   *.* :

MTr7            ----------     457
MTr6            GAPAARTA--     471
MTr4            GAPAARTA--     471
MTr5            GAPAARTA--     471
MTr3            DTAAARTA--     471
MTr1            DAPAARTA--     471
MTr2            DTAAARTA--     471
EGT7            GQDEQPAVQR     463
EGT6            ATP-------     475
EGT4            ATP-------     475
EGT5            ATP-------     475
EGT3            PTHSA-----     475
EGT2            PTPSA-----     475
EGT1            PTPSA-----     475
EGT1A           PTPSA-----     475
```

Figure S10. Alignment of polyene extending glycosyltransferases and their corresponding mycosaminyltransferases (see Table S2). Residues that differ between mycosaminyltransferases and extending glycosyltransferases are coloured red. Residues that differ between mannose-specific and N-acetyl glucosamine-specific extending glycosyltransferases are coloured blue.

```
EGT8      MDATRRPILFSCTESAGVFNPHVLLAGELAGRGVDDLWFATDENRRGDVEAASDKTDVRF       60
EGT9      --MTRKPILFCCTQSTGLMNSSLSVAGELARRGVPDLWFATDENRRVEVEKLAARSEVGF       58
EGT7      -MTSHRPILFCCTHSTGQATSSLVLAGEFAARGVKNLWFASDDNHRAAVAGLSGASEVGF       59
             :::****.**.*:*  .  : :***:* *** :****:*:*:*  *   :  ::* *

EGT8      LSMGEVNPRDVVTNYDDRTYRAVTQPEPFKAYLARVKQSLDLDGHYARYHRLAAAVDEIR       120
EGT9      VSLGDVDPALAATMWDEPTYRAVTGRSRFVAYRARVRAGMDAGYVADKYSRLSEAVARIK       118
EGT7      VSTGPVNPRVAPTMWDDETYRSITQRSRWKGNRARLRQLMDPVHHADRFRQLDAAVQRIQ       119
          :* * *:*  . * :*: ***::*  . : .  **::  :*      :: :*  ** .*:

EGT8      PALLVINNLNMHAVHLAVTRNIPFVLVAPCLPSDVLKATLPPEYPAPGTGLPLRMDARQQ       180
EGT9      PALMVINSLCHHAFLVAITHRIPFVITAPFLPSDLCQANLPKGFPVPQSGMPRDMTRAQR       178
EGT7      PALMVINNLCIHGIQVAMKRGVPYVITGSSLASDIWQFDLPDDYPVPYSGLPRRMNLRQR       179
          ***:***.*  *.. :*:.: :*:*:..  * **: :  **  :*.* :*:*  *   *:

EGT8      QANRRFRRRKRTMFLDGTVLKHSIAFEKARKAAGIDAKVLNSAAIMDQVELILCFSVFGL       240
EGT9      FAHWLFRLRMRTLVLDPVILRKAVAYGKAMDRLGVDRAARPQNAPFDAAEMILCFSLLEL       238
EGT7      IGNRLFRHRNLLSMLDPALLRTVGAMLKATDELGLDRRQLQQRAWMEEAELVLCFSVFGL       239
           .:  ** *    .** .:*:   *  **  .  *:*     . * :: .*::****:: *

EGT8      EYPFDATDKLRMLGAAVPPVPDGPAD-DVVTWLDAHRSVVFVSFSSGMRLTEAEIVAVTE       299
EGT9      DYPFPVPDKLHMLGTMVPPLPETSGDDDLTRWLDAQESVVYVAFGTITRLTRDEVAAMVE       298
EGT7      DYPFPHPPKLQMVGAMVPPLPQNPVPGETDRWLDAHPSVVFIAFGTITRLTKAEVRAVVD       299
          :***    **:*:*: ***:*:     :   ****: ***:::*.:  ***. *: *:.:

EGT8      AARRLGD-HSVLWKLPPSQQALL---GDLPPNLRVVDAVRSPAEVLAHPHVRAFVNHGGS       355
EGT9      VARRLGDRHQVLWKLPREQQAFLPPAGELPANLRIESWLPSQYDVLAHRNVKVFFNHAGS       358
EGT7      VARRLGERHHVLWKLPREQQRFLPPAAELPANLRVEDWLPSQYDVLAHSNVRVFFGHGGN       359
          .*****: * ****** .** :*   .:** ***: . : *  :**** :*:.*..*.*.

EGT8      NSVNQGLYFGRPLLIRPLWLDGRDHAVRVADSGVGLTVD---ALTADALHTALTRLLTEP       412
EGT9      NSFHEGLYFGKPLLSRPLWLDCYDHAVRAVDSGAGLTVDRPDTVDPDDVHHKLRRLLEED       418
EGT7      NSFHEGIYFGKPSLVRPLWFDCLDHAVRAVDSGVGLSVA-PGTMDPAEIHSKLTALLDDT       418
          **.::*:***:* * ****:*  *****..***.**:*    ::    :*  *  ** :

EGT8      SFTERADHLGRAQRSAGGVPAAADLILETPSMR------------       445
EGT9      SFRERAGYFSELQRRTGGVRAAADLILGSRALS------------       451
EGT7      SFRARAEHFAQIQHEAGGVRRAADLILRCAAVTSAGQDEQPAVQR       463
          **  ** ::.. *: :***  ******   ::
```

Figure S11. Alignment of PegA (EGT7) with *Crypto. arvum* (EGT8) and *Amyc. suaedae* (EGT9) GTs.

PKS1 (EXG82140.1)
KS^{Q}-ATL$_{MM}$-ACP-KS1-AT1$_{MM}$-KR1$_{B2}$-ACP1-KS2-AT2-KR2$_{B}$-ACP2-KS3-AT3$_{MM}$-DH3-KR3$_{B1}$-ACP3-KS4-AT4$_{MX}$-KR4$_{C1}$-ACP4-KS5-AT5$_{MX}$-KR5$_{A2}$-ACP5-KS6-AT6$_{MM}$-KR6$_{B1}$-ACP6-KS7-AT7$_{MM}$-KR7$_{B2}$-ACP7-KS8-AT8$_{MM}$-KR8$_{B2}$-ACP8-KS9-AT9$_{MM}$-KR9$_{A1}$-ACP9

PKS2
(EXG82141.1)
KS10-AT10-KR10$_{B}$-ACP10-KS11-AT11$_{MX}$-DH11-KR11$_{B1}$-ACP11-KS12-AT12$_{MM}$-KR12$_{A1}$-ACP12-KS13-AT13-DH13-KR13$_{B1}$-ACP13-KS14-AT14-DH14-KR14$_{B1}$-ACP14-KS15-AT15

(EXG82142.1)
DH15-KR15$_{B1}$-ACP15-KS16-AT16-DH16-KR16$_{B}$-ACP16-KS17-AT17-DH17-KR17$_{B1}$-ACP17-KS18-AT18-DH18-KR18$_{B1}$-ACP18-KS19-AT19-DH19-KR19$_{B1}$-ACP19

PKS3 (EXG82135)
KS20-AT20-DH20-KR20$_{B}$-ACP20-KS21-AT21-KR21$_{A}$-ACP21-KS22-AT22$_{MM}$-KR22$_{A2}$-ACP22-KS23-AT23-KR23$_{B}$-ACP23-KS24-AT24-KR24$_{C}$-ACP24-KS25-AT25-KR25$_{B}$-ACP25

PKS4

(EXG82136.1)
KS26-AT26-KR26$_{B}$-ACP26-KS27-AT27-KR27$_{B}$-ACP27-KS28-

(EXG82137.1)
AT28-DH28-KR28$_{B}$-ACP28

PKS5 (EXG82138.1)
KS29-AT29-DH29-ER29-KR29$_{B}$-ACP29-KS30-AT30-DH30-ER30-KR30$_{B}$-ACP30-KS31-AT31-DH31-KR31$_{B}$-ACP31-TE

Figure S12. Domain compositions of *Cryptosporangium arvum* DSM 44712 PKS proteins. The *Cryptosporangium arvum* DSM 44712 genome has accession number JFBT01000001. The genes in the polyene BGC are listed in Excel file 1 sheet E. The motifs relevant for structure prediction are listed in Excel file 2 sheet 5.

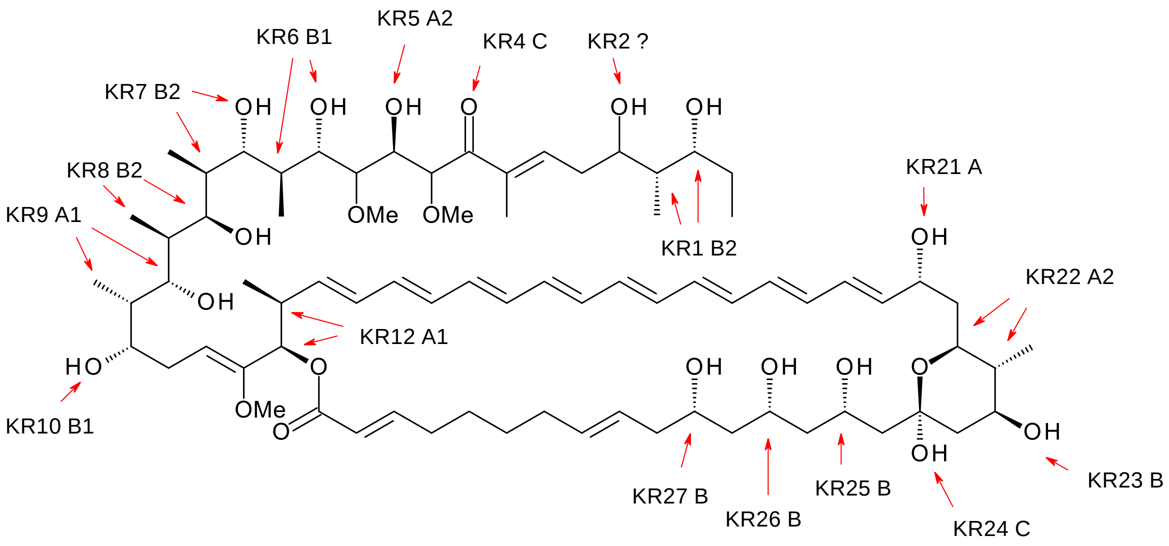

Figure S13. Stereostructure predicted for *Cryptosporangium arvum* DSM 44712 octaene.

PKS1 (WP_130475492.1)
ACP$_{L}$-KS1-ATL$_{B}$-AT1$_{EM}$-ACP1

PKS2 (WP_130475491.1)
KS2-AT2$_{MM}$-DH2-ER2-KR2$_{B}$-ACP2

PKS3 (WP_130475490.1)
KS3-AT3-DH3-KR3$_{B}$-ACP3-KS4-AT4-DH4-KR4$_{B}$-ACP4-KS5-AT5-KR5$_{A}$-ACP5-KS6-AT6$_{MM}$-DH6^{0}-KR6$_{B}$-ACP6

PKS4 (WP_130475489.1)
KS7-AT7-DH7-KR7$_{B}$-ACP7-KS8-AT8$_{MM}$-DH8-KR8$_{B}$-ACP8-KS9-AT9$_{MM}$-DH9-KR9$_{B}$-ACP9

PKS5 (WP_130475499.1)
KS10-AT10-DH10-KR10$_{B}$-ACP10-KS11-AT11-KR11$_{A}$-ACP11-KS12-AT12$_{MM}$-KR12$_{A2}$-ACP12-KS13-AT13-KR13$_{B}$-ACP13-KS14-AT14-KR14$_{(deleted)}$-ACP14

PKS6 (WP_130475498.1)

KS15-AT15-DH15-KR15$_B$-ACP15-KS16-AT16-DH16-ER16-KR16$_B$-ACP16

PKS7 (WP_130475497.1)
KS17-AT17-DH17-KR17$_B$-ACP17-TE

Figure S14. Domain compositions of the seven *Amyc. suaedae* PKS proteins. The *Amycolatopsis suaedae* genome has accession number NZ_SFCC01000005, NZ_SFCC01000018. The genes in the polyene BGC are listed in Excel file 1 sheet F. The motifs relevant for structure prediction are listed in Excel file 2 sheet 6.

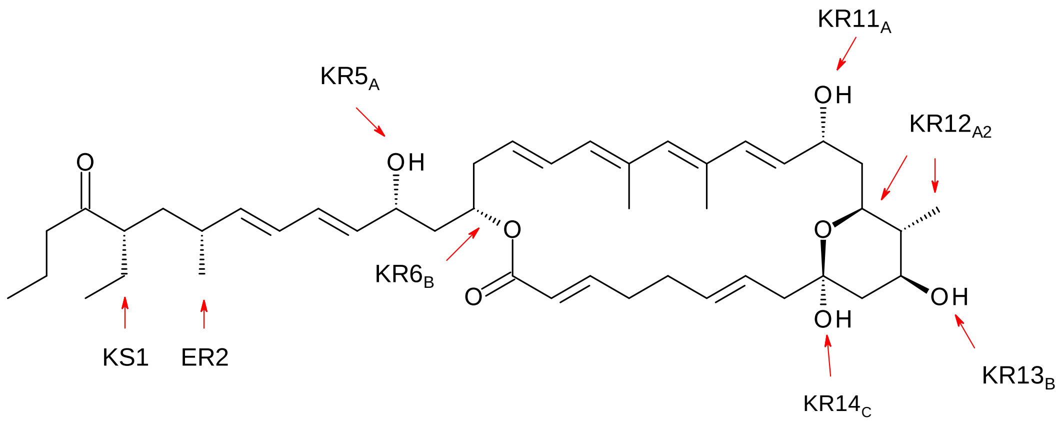

Figure S15. Stereostructure predicted for *Amyc. suaedae* polyketide.

PKS1 (MBB5070959.1)
KS$_0$-AT$_0$-ACP$_0$

PKS2 (MBB5070958.1)
KS1-AT1$_{MM}$-KR1$_{A2}$-ACP1-KS2-AT2$_{MM}$-KR2$_{A1}$-ACP2

PKS3 (MBB5070957.1)
KS3-AT3-DH3-KR3$_B$-ACP3-KS4-AT4-DH4-KR4$_B$-ACP4-KS5-AT5-DH5-KR5$_B$-ACP5-KS6-AT6-DH6-KR6$_B$-ACP6

PKS4 (MBB5070970.1)
KS7-AT7-DH7-KR7$_B$-ACP7-KS8-AT8-KR8$_A$-ACP8-KS9-AT9-KR9$_{A2}$-ACP9-KS10-AT10-KR10$_B$-ACP10-KS11-AT11-KR11$_C$-ACP11-KS12-AT12-KR12$_B$-ACP12

PKS5 (MBB5070969.1)
KS13-AT13-DH13-ER13$_R$-KR13$_B$-ACP13

PKS6 (MBB5070968.1)
KS14-AT14-DH14-KR14$_B$-ACP14-TE

Figure S16. Domain compositions of *Sacc. gloriosae* PKS proteins. The *Sacc. gloriosae* genome has accession number JACHIV010000001. The genes in the polyene BGC are listed in Excel file 1 sheet G. The motifs relevant for structure prediction are listed in Excel file 2 sheet 7.

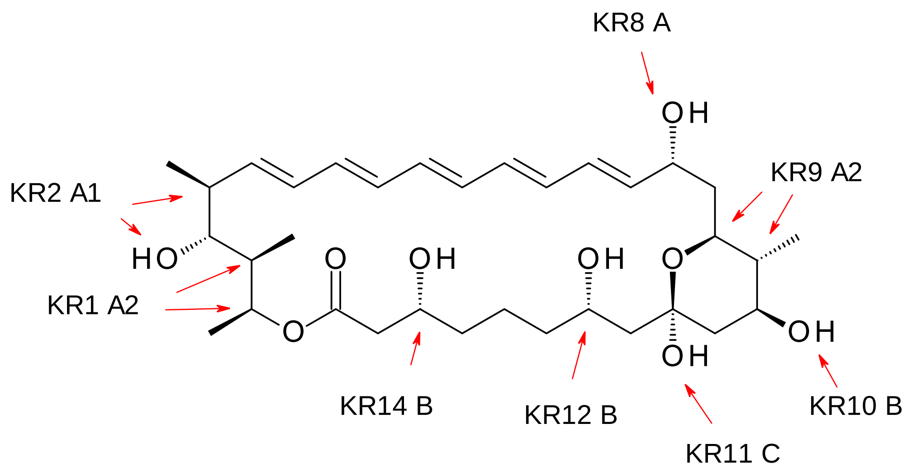

Figure S17. Predicted stereostructure of *Sacc. gloriosae* polyketide.

Amyc. cihanbeyliensis contig 1

PKS1 (WP_141995325.1)
ACP0-KS1-AT0$_{Ac}$-AT1$_{MM}$-KR1$_{B}$-ACP1

PKS2 (WP_141995326.1)
KS2-AT2$_{M}$-DH2^{0}-KR2$_{B}$-ACP2-KS3-AT3$_{MM}$-DH3-ER3-KR3$_{B}$-ACP3

PKS3 (WP_141995337.1)
KS4-AT4-DH4-KR4$_{B}$-ACP4-KS5-AT5-DH5-KR5$_{B}$-ACP5-KS6-AT6$_{MM}$-KR6$_{A2}$-ACP6-KS7-AT7-KR7$_{B}$-ACP7

PKS4 (WP_141995338.1)
KS8-AT8-DH8-KR8$_{B}$-ACP8-KS9-AT9$_{MM}$-DH9-KR9$_{B}$-ACP9-KS10-AT10-DH10-KR10$_{B}$-ACP10

Amyc. cihanbeyliensis contig 2

PKS5 (WP_142002652.1)
KS11-AT11-DH11-KR11$_{B}$-ACP11-KS12-AT12-KR12$_{A}$-ACP12-KS13-AT13$_{MM}$-KR13$_{A2}$-ACP13-KS14-AT14-KR14$_{B}$-ACP14-KS15-AT15-KR15$_{C}$-ACP15

Figure S18. Domain compositions of the *Amyc. cihanbeyliensis* PKS proteins for which sequence data are available. The *Amyc cihanbeyliensis* genome has accession number NZ_VFML01000001, NZ_VFML01000002. The genes in the polyene BGC are listed in Excel file 1 sheet H. The motifs relevant for structure prediction are listed in Excel file 2 sheet 8.

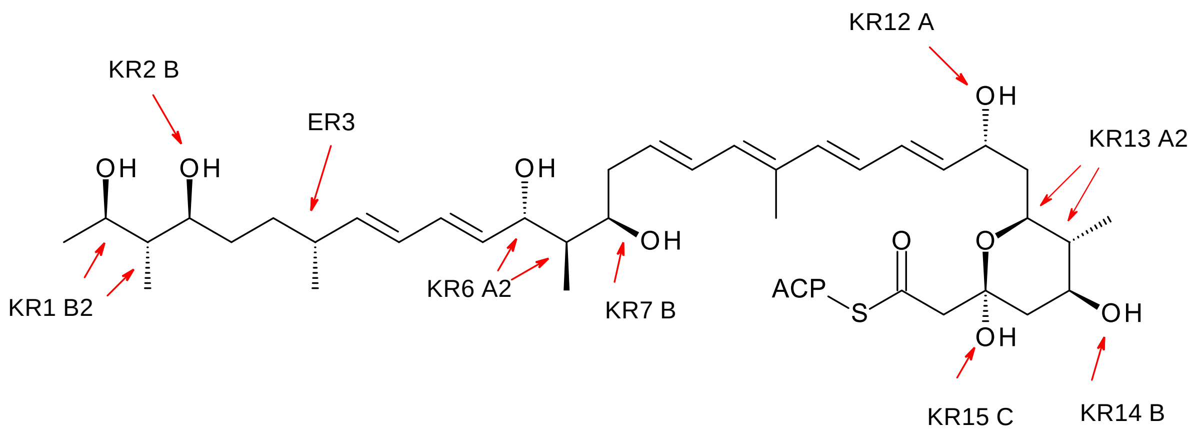

Figure S19. Fragment of *Amyc. cihanbeyliensis* polyene.

PKS1 (WP_185005834.1)
ACP-KS-ATL$_{MM}$-AT1$_{MM}$-KR1$_{B}$-ACP

PKS2 (WP_185005833.1)
KS-AT2$_{M}$-DH2-KR2$_{B}$-ACP-KS-AT3$_{MM}$-DH3-ER3-KR3$_{B}$-ACP

PKS3 (WP_185005832.1)
KS-AT4$_{MM}$-DH4^{0}-KR4$_{B}$-ACP-KS-AT5$_{M}$-KR5$_{B}$-ACP-KS-AT6$_{M}$-ACP-KS-AT7$_{MM}$-KR7$_{A}$-ACP

PKS4 (WP_185005831.1)
KS-AT8$_{MM}$-DH8^{0}-KR8$_{B}$-ACP

PKS5 (WP_185005830.1)
KS-AT9$_{M}$-DH9-KR9$_{B}$-ACP-KS-AT10$_{MM}$-DH10-KR10$_{B}$-ACP-KS-AT11$_{M}$-DH11-KR11$_{B}$-ACP-KS-AT12$_{M}$-DH12-KR12$_{B}$-ACP

PKS6 (WP_185005849.1)
KS13-AT13$_{M}$-DH13-KR13$_{B}$-ACP13-KS14-AT14$_{M}$-KR14$_{A}$-ACP14-KS15-AT15$_{MM}$-KR15$_{A}$-ACP15-KS16-AT16$_{M}$-KR16$_{B}$-ACP16-KS17-AT17$_{M}$-KR17$_{C}$-ACP17

PKS7 (WP_185005835.1)
KS18-AT18$_{M}$-DH18^{0}-KR18$_{B}$-ACP18-KS19-AT19$_{M}$-DH19^{0}-KR19$_{B}$-ACP19-KS20-AT20$_{M}$-DH20-KR20$_{B}$-ACP20

PKS8 (WP_185005824.1)
KS21-AT21$_{MM}$-DH21-ER21-KR21$_{B}$-ACP21-TE

Figure S20. Domain compositions of the eight *C. cryophila* PKS proteins. The genes present are listed in Excel file 1 Sheet I. The relevant motifs from the 21 extension modules are listed in Excel file 2 Sheet 9. PKS1 (Protein ID WP185005834.1) is 42% identical to MxaF protein from *Stigmatella aurantiaca*, which functions in mxyalamid biosynthesis. The two proteins have the same domain composition [ACP-KS-AT$_{MM}$-AT$_{MM}$-KR$_{B}$-ACP] (Silakowski et al., 2001) and both are likely to function as loading module and first extension module. In the MxaF protein the first AT domain loads a propionyl starter onto the N-terminal ACP and the second AT domain loads a methylmalonyl extender onto the C-terminal ACP. The KS domain catalyses the first condensation.

Silakowski, B.; Nordsiek, G.; Kunze, B.; Blöcker, H.; Müller, R. Novel Features in a Combined Polyketide Synthase/Non-Ribosomal Peptide Synthetase: the Myxalamid Biosynthetic Gene Cluster of the Myxobacterium *Stigmatella aurantiaca* Sga1511. *Chem. Biol.* **2001**, *8*(1), 59-69

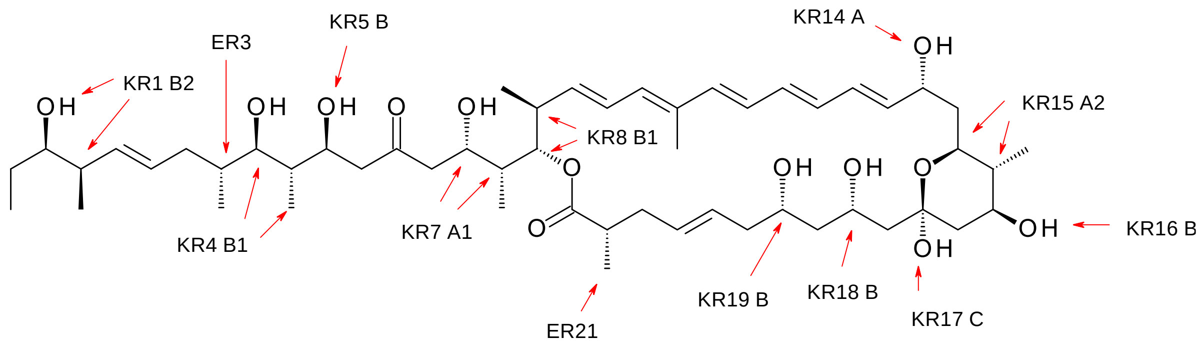

Figure S21. Predicted structure of cyclised *C. cryophila* polyketide.

KS-AT-DH-KR$_{B}$-ACP-KS-AT-KR$_{A}$-ACP-KS-AT$_{MM}$-KR$_{A2}$-ACP-KS-AT-KR$_{B}$-ACP-KS-AT-KR$_{C}$-ACP

Figure S22. Domain composition of *Amyc. antarctica* PKS protein. The *Amycolatopsis antarctica* genome has accession number NZ_NKYE01000003. The genome sequence is incomplete but encodes a pentamodular polyene PKS protein capable of synthesising the mycosaminylation site. There are biosynthetic genes for GDP-mycosamine and an AmphDI (mycosaminyltransferase GT) OZM74150.1. The genes in the polyene BGC are listed in Excel file 1 sheet J. The motifs relevant for structure prediction are listed in Excel file 2 sheet 10.

PKS 1 MBB4911532.1
ACP$_{L}$-KS-AT$_{L}$-AT1$_{MM}$-ACP1

PKS 2 MBB4911531.1
KS2-AT2$_{MM}$-DH2-ER2-KR2$_{B1}$-ACP2

PKS 3 MBB4911530.1
KS3-AT3-DH3-KR3$_{B}$-ACP3-KS4-AT4-DH4-KR4$_{B}$-ACP4-KS5-AT5$_{MM}$-KR5$_{A2}$-ACP5-KS6-AT6-DH6^{0}-KR6$_{B}$-ACP6

PKS 4 MBB4911529.1
KS7-AT7-DH7-KR7$_{B}$-ACP7-KS8-AT8-DH8-KR8$_{B}$-ACP8-KS9-AT9-DH9-ACP9

PKS 5 MBB4911539.1
KS10-AT10-DH10-KR10$_{B}$-ACP10-KS11-AT11-KR11$_{A}$-ACP11-KS12-AT12$_{MM}$-KR12$_{A2}$-ACP12-KS13-AT13-KR13$_{B}$-ACP13-KS14-AT14-KR14$_{B}$-ACP14

PKS 6 MBB4911538.1
KS15-AT15-DH15-ER15-KR15$_{B}$-ACP15-KS16-AT16-DH16-ER16-KR16$_{B}$-ACP16

PKS 7 MBB4911537.1
KS17-AT17-DH17-KR17$_{B}$-ACP17-TE

Figure S23. Domain compositions of the seven *A. algeriensis* PKS proteins. The *Actinophytocola algeriensis* genome has accession number JACHJQ010000010. The genes in the polyene BGC are listed in Excel file 1 sheet K. The motifs relevant for structure prediction are listed in Excel file 2 sheet 11.

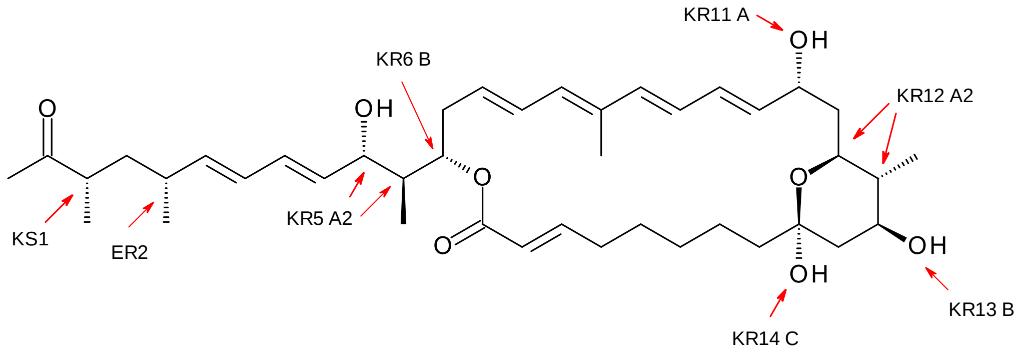

Figure S24. Predicted structure of *A. algeriensis* polyketide.
